# Repositioning of drugs for Parkinson’s disease and pharmaceutical nanotechnology tools for their optimization

**DOI:** 10.1186/s12951-022-01612-5

**Published:** 2022-09-15

**Authors:** Héctor Hernández-Parra, Hernán Cortés, José Arturo Avalos-Fuentes, María Del Prado-Audelo, Benjamín Florán, Gerardo Leyva-Gómez, Javad Sharifi-Rad, William C. Cho

**Affiliations:** 1 Departamento de Farmacología, Centro de Investigación Y de Estudios Avanzados del Instituto Politécnico Nacional (CINVESTAV-IPN), Ciudad de Mexico, Mexico; 2 Departamento de Farmacia, Facultad de Química, Universidad Nacional Autónoma de México, Ciudad de Mexico, Mexico; 3 Laboratorio de Medicina Genómica, Departamento de Genómica, Instituto Nacional de Rehabilitación Luis Guillermo Ibarra Ibarra, Ciudad de Mexico, Mexico; 4 Departamento de Fisiología, Biofísica & Neurociencias, Centro de Investigación y de Estudios Avanzados del Instituto Politécnico Nacional (CINVESTAV-IPN), Ciudad de Mexico, Mexico; 5 Escuela de Ingeniería Y Ciencias, Tecnologico de Monterrey, Campus Ciudad de México, C. Puente 222, 14380 Ciudad de México, Mexico; 6 Facultad de Medicina, Universidad del Azuay, Cuenca, Ecuador; 7 Department of Clinical Oncology, Queen Elizabeth Hospital, Kowloon, Hong Kong

**Keywords:** Drug repositioning, Drug repurposing, Parkinson’s disease, Nanoparticles, Nanocarriers, Pharmaceutical nanotechnology

## Abstract

Parkinson’s disease (PD) significantly affects patients’ quality of life and represents a high economic burden for health systems. Given the lack of safe and effective treatments for PD, drug repositioning seeks to offer new medication alternatives, reducing research time and costs compared to the traditional drug development strategy. This review aimed to collect evidence of drugs proposed as candidates to be reused in PD and identify those with the potential to be reformulated into nanocarriers to optimize future repositioning trials. We conducted a detailed search in PubMed, Web of Science, and Scopus from January 2015 at the end of 2021, with the descriptors “Parkinson’s disease” and “drug repositioning” or “drug repurposing”. We identified 28 drugs as potential candidates, and six of them were found in repositioning clinical trials for PD. However, a limitation of many of these drugs to achieve therapeutic success is their inability to cross the blood–brain barrier (BBB), as is the case with nilotinib, which has shown promising outcomes in clinical trials. We suggest reformulating these drugs in biodegradable nanoparticles (NPs) based on lipids and polymers to perform future trials. As a complementary strategy, we propose functionalizing the NPs surface by adding materials to the surface layer. Among other advantages, functionalization can promote efficient crossing through the BBB and improve the affinity of NPs towards certain brain regions. The main parameters to consider for the design of NPs targeting the central nervous system are highlighted, such as size, PDI, morphology, drug load, and Z potential. Finally, current advances in the use of NPs for Parkinson's disease are cited.

## Introduction

Neurological disorders are considered a leading cause of disability [1]. Parkinson’s disease (PD) is the second most frequent neurodegenerative disease worldwide only after Alzheimer’s disease [2] and has overgrown in recent years. In this respect, 6.1 million people were registered with PD worldwide in 2016, and it caused 211,296 deaths only in that year [3]. PD affects approximately 1% of the population over 60 years old; the prevalence is around 1.4 times higher in men than in women, increasing with the population aging [3, 4]. Since life expectancy has been increasing in the last years, patients with PD are expected to increase. According to a PD’s global burden study, the number of PD patients will be approximately 13 million in 2040 [3]. Thus, it is expected an enormous social and economic burden for both health systems and the people in charge of patient care.

Notwithstanding advances in understanding PD, currently available therapies are symptomatic but do not stop the disease’s progression [5]. Besides, drugs used for PD treatment produce side effects that can be very serious and disabling, such as dyskinesias. Therefore, it is necessary to implement new drug search and discovery methods that offer more effective and safer treatment alternatives. In addition to these worldwide efforts, drug repositioning has been applied, which reduces costs and research times compared to the traditional de novo drug development strategy [6].

Currently, numerous drugs are under study, of which promising results have been reported [7–12]. However, despite these results, some drugs present limitations to achieving therapeutic success, such as bioavailability problems and reduced capacity to cross the blood–brain barrier (BBB). This review analyzes information on drugs proposed for repositioning in the treatment of PD in the last six years. Those drugs with limitations in their bioavailability and targeting to the brain are identified, and pharmaceutical nanotechnology strategies are proposed to optimize future repositioning studies of these drugs. The most effective treatment is levodopa, but its benefits are compromised by unpredictable absorption and extensive peripheral metabolism, leading to motor fluctuations and loss of efficacy [13].

## Parkinson’s disease

Although the exact etiology of PD is not known, the risk of developing it seems to be determined by biological factors (such as age), genetic factors (such as the presence of specific polymorphisms or mutations), and environmental factors (e.g., exposure to pesticides such as rotenone and paraquat) [14–16]. PD is characterized by causing progressive degeneration of dopaminergic neurons of the substantia nigra pars compacta (SNpc) that project towards the striatum and other brain nuclei [17]. Another of its main characteristics is the presence of Lewy bodies, intraneuronal inclusions formed by insoluble aggregates of abnormally folded alpha-synuclein protein [18].

The physiological basis of PD is dysfunction of the basal ganglia due to the loss of dopamine (DA), its central modulator [18]. DA is a neurotransmitter that is produced in the neurons of the substantia nigra; it is released into the striatum to execute uniform and deliberate movements [14]. In this context, the loss of DA produces brain nerve activity abnormalities that cause impaired movement. Degeneration of dopaminergic neurons occurs mainly in the SNpc, in which DA is usually synthesized and released to the brain regions that regulate movement. However, some reports estimate that motor symptoms appear when more than half (between 50 and 70%) of dopaminergic neurons of the SNpc are degraded [19]. This delayed effect on motor symptoms is due to the striatum downstream of the SNpc triggering compensatory mechanisms, which can respond to a certain degree, as eventually, neurons in the striatum also begin to die [20]. Both genetic factors (in familial PD) and environmental factors (in sporadic PD) converge on specific pathways, including mitochondrial dysfunction, oxidative stress, protein aggregation, impaired autophagy, and neuroinflammation, leading to the clinical manifestations of PD [21, 22]. The activation of the c-Abl protein (Abelson tyrosine kinase) is related to various pathogenic pathways that could lead to neuronal death in response to oxidative stress in PD. Oxidative stress has been considered a critical process in sporadic PD and familial PD. When activated, the c-Abl protein acts as an oxidative stress sensor that can generate multiple downstream signals that lead mainly to parkin inactivation, p38α activation, and α-synuclein phosphorylation. The inactivation of parkin causes the accumulation of pathogenic substrates (PARIS and AIMP2), leading to neuronal death, p38α activation, and α-synuclein phosphorylation, which are potentially related to cytotoxicity and neuronal death [23]. In this respect, the inhibition of the c-Abl protein could represent a powerful therapeutic target for PD [24, 25].

### Burden of Parkinson’s disease

PD symptoms are motor and non-motor and can affect the patient’s quality of life (QoL) since it is a highly disabling disease. The disabling and progressive effect in a patient with PD requires other people (caregivers) to carry out daily activities. The burden for these caregivers is a broad topic since they must provide emotional, physical, and social support, and as PD progresses, care must be more rigorous, so much so that the QoL of caregivers can be seriously affected, developing stress, anxiety, depression, and other health problems [26]. PD’s economic burden is borne by the patient and their families, although it also represents a significant burden for each country’s health systems. In a study conducted in the US, it is estimated that at least until 2017, PD represented a total economic burden of $51.9 billion; the total burden includes direct medical costs of $25.4 billion and $26.5 billion in indirect and non-medical costs [27]. In that same study, a total economic burden is projected for 2037, exceeding 79 billion dollars. Considering that the greater the disease’s progression, the greater the economic cost, we believe that new interventions are necessary and urgent to delay the progression of the disease and alleviate the burden of symptoms, and in this way, the future burden of PD could be reduced.

### Treatment for Parkinson’s disease

At present, no treatment cures or stops PD progression; however, various therapeutic options are limited to partially alleviating the signs and symptoms, allowing patients to improve their QoL by less for a time, which depends on the disease’s progression. Treatment options include surgical and pharmacological therapies.

Surgical treatment: Deep brain stimulation; Focused ultrasound; Cell replacement therapies. Many patients with moderate to advanced disease resort to this type of treatment in conditions where they do not respond to pharmacological medication. However, in this type of treatment, essential aspects such as cost and risk must be considered, which are generally high. Its success depends on the appropriate selection of patients and the surgeon’s experience and skill to optimize results and minimize complications [28]. In this respect, it is preferable to use less invasive, cheaper, and less risky therapies, so pharmacological therapies are used as first-choice treatments.

Pharmacological treatment: Most of these drugs have focused on restoring neuronal dopaminergic transmission [18]. However, drugs targeting the glutamatergic, noradrenergic, serotonergic, and cholinergic systems are also being used, playing a fundamental role in the basal ganglia circuits. Nevertheless, these do not stop the progression of the disease if they have managed to improve the characteristic symptoms.

Treatment of motor symptoms:

*Levodopa* It is an oral precursor of DA and is to date considered the “gold standard” of PD treatment [29], and it is the most effective drug for the treatment of motor manifestations [18].

*Anticholinergics* Like trihexyphenidyl and benztropine, which antagonize acetylcholine’s effects at postsynaptic muscarinic receptors to striatal interneurons, they are used primarily to reduce tremor and have no effect on bradykinesia [28].

*Antiglutamatergics* Amantadine (glutamate/NMDA receptor antagonist). A prolonged-release formulation of amantadine, administered before bedtime, improves dyskinesia and motor fluctuations [28].

*Monoamine oxidase inhibitors (MAOIs)* Include selegiline, rasagiline, and safinamide, which, although more frequently used in mild and early PD, these MAOIs are also effective in patients with moderately advanced PD with levodopa-related motor complications [28, 30].

*Dopamine agonists* Like the non-ergot derivatives, the most common of which include pramipexole, ropinirole, rotigotine, and apomorphine, can be used as monotherapy for motor symptoms or add-on therapy when symptoms are not controlled by levodopa or when motor fluctuations are present [31].

*Catechol-O-methyl transferase inhibitors (COMTI)* Like entacapone, tolcapone, and opicapone, which block the degradation of peripheral levodopa and the central degradation of levodopa and DA, increasing the central levels of these [28].

*Adenosine A2 receptor antagonist* Istradefylline (Nourianz) is an add-on treatment to levodopa/carbidopa in PD patients who experience inactive episodes [32].

Treatment of non-motor symptoms:

There are a wide variety of non-motor symptoms, including depression, anxiety, apathy, psychosis, to name a few, and each of them must be treated specifically. As an example, we cite the treatment of some of the common non-motor symptoms. Such as donepezil, rivastigmine, and memantine provide a modest benefit in patients with PD-associated dementia [28]. Alternatively, pimavanserin, a serotonin inverse agonist with a high affinity for the 5-HT2A receptor, was approved by the Food and Drug Administration (FDA) in 2016 to treat hallucinations and delusions associated with PD [33].

### Complications related to drug treatment

Currently available treatment for PD can significantly improve symptoms. However, with prolonged use, the efficacy of the drugs tends to decrease, and complications related to the treatment appear. Patients with PD show significant variability in response to drugs in terms of efficacy and adverse effects. Some studies have associated interindividual variability in response to treatment with genetic and environmental factors [34–37]. Currently, many studies focus on the genetic variability in response to levodopa, the main drug used in clinical practice to treat PD. Some patients have been found to develop low toxicity at high doses of dopaminergic treatment, while others have severe side effects [38]. Many patients respond positively to treatment with levodopa for many years, while others fail to achieve a therapeutic effect in a few years. Evidence shows that around 40% of patients develop motor complications within the first 4 to 6 years of treatment with levodopa [39, 40]. Although several genes have been studied, only some of them have been investigated in large cohorts, such as the DRD2, COMT, and SLC6A3 gene polymorphisms [34]. Some authors associate the decreased efficacy of drugs with the worsening of the disease and the presence of dopaminergic brain lesions and underlying non-dopaminergic ones [41]. However, it is believed that other genes and factors could influence the variability in response to drugs and the decrease in clinical efficacy.

Other complications related to pharmacological treatment are motor and motor and non-motor fluctuations, dyskinesias, impulse control disorder (ICD), dopaminergic dysregulation syndrome (DDS), DA agonist withdrawal syndrome (DAWS), and levodopa withdrawal syndrome [42]. These complications associated with pharmacological treatment can be treated specifically, as is the case of Levodopa-induced dyskinesia (LID), which is reduced by concomitant administration of amantadine, which is currently the main drug for its treatment [28]. These side effects can be even more disabling than those of the disease itself in the early stages. Among the most recurrent adverse effects, in general, we find; for DA agonists, drowsiness, nausea, vomiting, dizziness, swelling of the legs, and sweating; for COMTI, dyskinesias, dizziness, nausea, vomiting, diarrhea, hallucinations, drowsiness, dry mouth, and abdominal pain; for MAO-B inhibitors, dizziness, drowsiness, heartburn, nausea, and weight loss. Also, other drugs can have serious adverse effects, such as amantadine (which can cause dizziness, hallucinations, confusion, constipation, hair loss, and possible exacerbations of heart failure) and trihexyphenidyl (which cause cognitive impairment, blurred vision, and urinary retention) [28].

As mentioned, levodopa remains the “gold standard” in treatment, and response to levodopa is even used as part of the diagnosis of PD. Unfortunately, treatment with levodopa after several years of use loses efficacy. Its prolonged use is associated with side effects, such as response fluctuations and LID, representing a significant disadvantage of continuous therapy [19]. Levodopa-related fluctuations have various clinical manifestations, and non-motor fluctuations generally precede and/or accompany motor fluctuations [43]. Among the motor fluctuations, the one with the earliest appearance is the “wearing-off” (end-of-dose deterioration) [44] which is characterized by a progressive shortening of the period between the intake of one dose and another levodopa; motor complications also include dyskinesias [42], different from those characteristics of the natural progression of PD. The ICD is pathological gambling, compulsive shopping, and hypersexual disorders, among other behavioral disorders associated with various drugs, such as levodopa, amantadine, and rasagiline [45, 46]. It is also essential to consider that not all clinical manifestations of PD are dopaminergic and that non-dopaminergic symptoms (such as sleep disorders, pain syndromes, mood disorders, and dementia) do not respond effectively to therapeutic possibilities currently available [42]. It is necessary to search for novel pharmacological options and approaches that optimize these drugs and promising search strategies, as is drug repositioning.

## Drug repositioning

The reuse of drugs already approved for different medical indications is becoming a compelling alternative for the scientific community and the pharmaceutical industry [47]. In the future, it could be of even greater interest to health financing organizations [48]. Drug repositioning consists of providing new therapeutic use to an existing drug and is a widely utilized strategy in recent years as an alternative to de novo drug development [6, 49]. De novo drug development has become increasingly challenging as approximately 90% of drugs fail during development in phase I clinical trials, making this process very risky, expensive, and requires a long time of experimentation [50]. Pharmaceutical companies increasingly explore drug repositioning with these risks and the low probability of success [49]. In recent years, it has been estimated that roughly a third of approvals are for drug reuse, and these repurposed drugs generate roughly around 25% of the annual revenue of the pharmaceutical industry [51, 52].

**Fig. 1:**
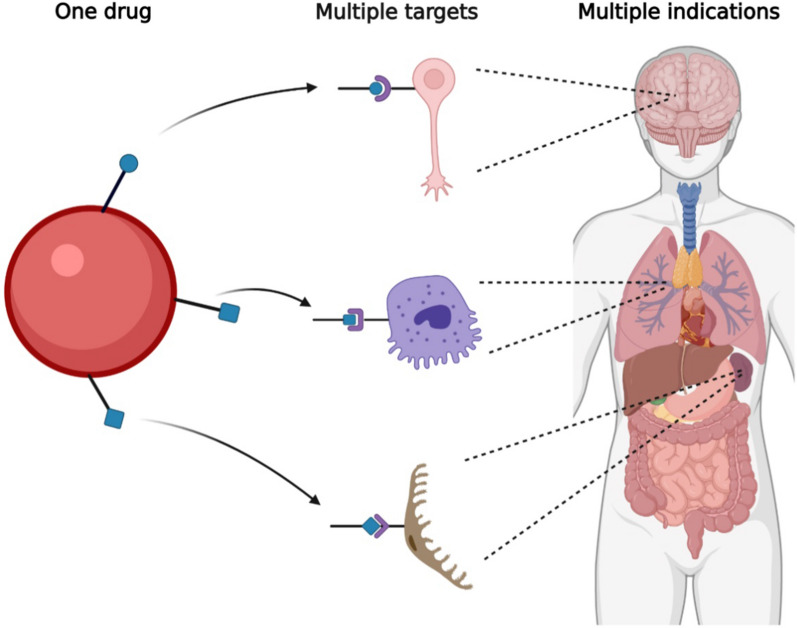
Drug repositioning and polypharmacology. A drug can have more than one active site, which allows the molecule to target different organs and gives rise to multiple therapeutic indications; this is known as polypharmacology and is used in the repositioning of drugs since there are drugs approved for a therapeutic indication for which its biological target is known, but with the potential to target other tissues and alleviate other pathologies

Drug repositioning takes advantage, considering that a single molecule can perform on multiple biological targets. This is known as polypharmacology [36]. It could be beneficial when additional targets are relevant or part of some other pathology for which the drug was not indicated initially (Fig. 1). Candidate drugs for repositioning, because they are already approved for use in humans, have exceeded regulatory standards, including preclinical, clinical, and post-marketing pharmacovigilance studies [52]. These studies allow the repositioning process to be in less time, with less economic investment, and with a greater probability of success. In this context, if the dose required for the new indication is the same as that used for the original symptom, part of the preclinical trials and even phase I clinical trials can be avoided [52]. However, new pharmacological safety studies will be necessary if the dose is higher or lower than that used in the original indication. Another advantage is that they can represent a desirable market for the pharmaceutical industry because when drugs are without intellectual protection, or their patent has expired, the possibility of obtaining a patent for the new indication opens up [53]. However, an ideal condition would be that the new indication requires non-marketed concentrations or that the drug requires a pharmaceutical reformulation, for example, in a novel form of administration that allows optimizing its use. By modifying the formulation of a reused drug, reformulators could obtain a novel patent since the invention would be considered a new composition of matter [54]. Historically, many drugs have been successfully repositioned; some examples of successful repositioning are Sildenafil, which was initially studied for use in hypertension and angina pectoris but has been repositioned to treat erectile dysfunction [50]. Rituxan was initially indicated for non-Hodgkin lymphoma and later approved for chronic lymphocytic leukemia and rheumatoid arthritis [55]. Currently, there are many candidate drugs for repositioning in multiple pathologies, which gives us hope in the face of the difficult task of finding new therapies for difficult-to-treat diseases, including neurodegenerative ones such as PD, which despite multiple efforts, has not yet developed a therapy capable of stopping or reversing the disease.

### Drug repositioning in Parkinson’s disease

**Fig. 2:**
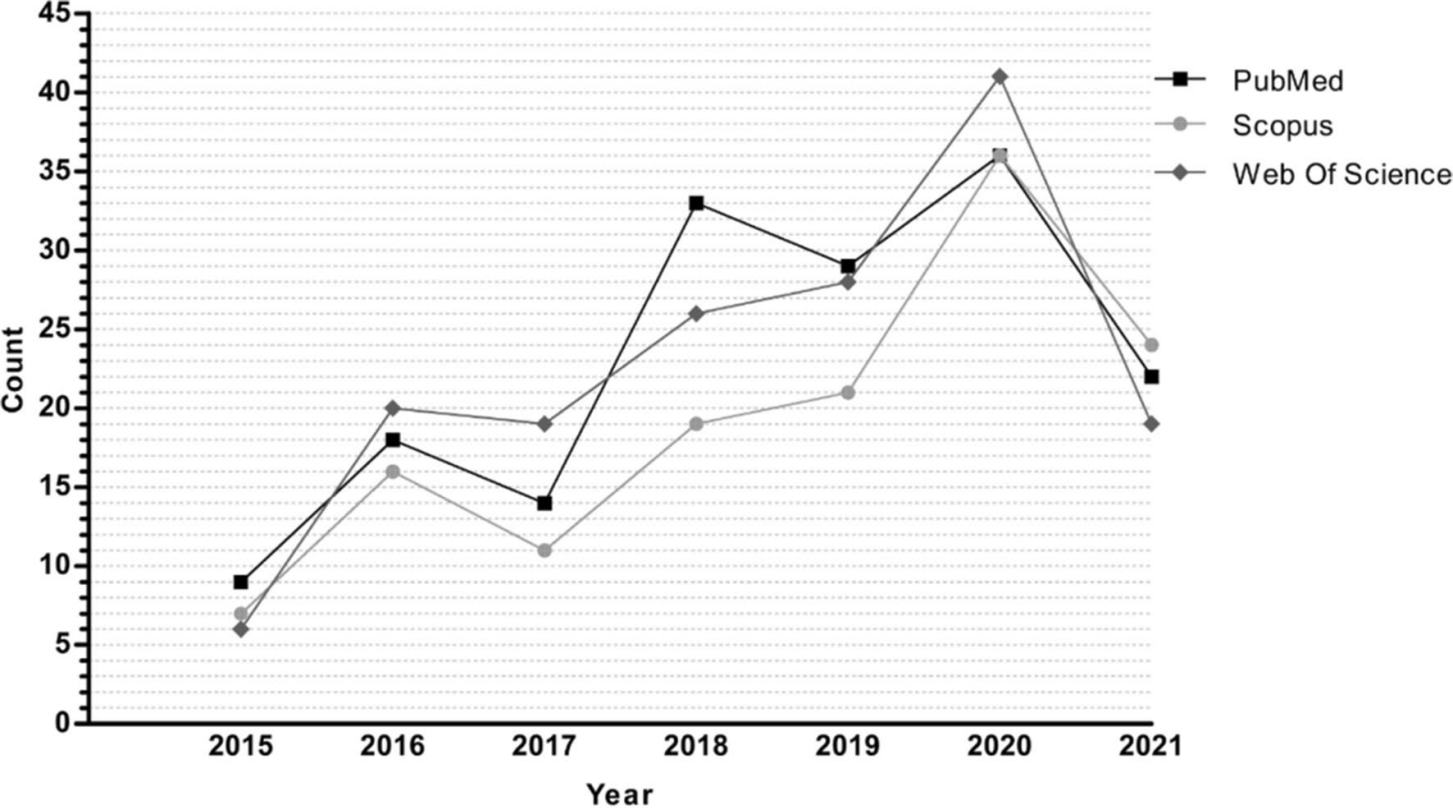
The number of publications indexed in PubMed, Web of Science, and Scopus that contain the terms “(Parkinson’s disease) and (drug repositioning or drug repurposing)” in the last years (01/2015–07/2021)

Some drugs have been successfully repositioned for PD treatment, such as amantadine, an antiviral repositioned to treat LID [14]. Currently, many drugs are in studies as candidates for repositioning for PD, some with encouraging results, but that need to be optimized for better efficacy. In this context, it is necessary to do bibliographic reviews that gather all this evidence, identifying areas of opportunity to propose improvements and solutions to possible limitations. We reviewed for the last few years (2015-present) to identify those drugs currently being proposed for repositioning in PD. The search was carried out in PubMed NCBI, Scopus, and Web of Science, with the search terms “(Parkinson’s disease) and (drug repositioning or drug repurposing),” and it was found that drug repositioning studies for PD have increased in recent years (Fig. 2). For example, for the PubMed search in the first two years of this period (2015–2017), only 41 publications were retrieved, and in the last four years (2018–2021), up to 98 publications, indicating an increase of more than 50% in articles related to at least our search conditions. The above demonstrates the expansion that the repositioning of drugs in PD is taking and the growing interest in the scientific community to communicate its results for the public benefit and find new treatment alternatives for PD.

Many are in early studies between the drug candidates for repositioning in silico or in vitro, and many others are in more advanced studies such as preclinical or clinical trials. In this analysis, 28 drugs were selected as proposed to be reused in PD treatment. The initial therapeutic indication, mechanism of action, the new suggested therapeutic indication, and the proposal for its new mechanism of action are highlighted in Table 1.

**Table 1:** Drugs proposed for repositioning in PD with suggested new mechanisms of action

Drugs	Initial therapeutic indication	Initial mechanism of action	Novel therapeutic indication suggested	Novel mechanism of action suggested	Model of evaluation	References
Exenatide	Type II diabetes mellitus	GLP-1 receptor agonist that promotes glucose-dependent insulin secretion	Neuroprotective in PD	Exerts neuroprotective effects through GLP-1 receptors, resulting in motor performance improvements, behavior, learning, and memory	Clinical trial, single-center, randomized, double-blind, placebo-controlled. The trial included 60 patients	[7]
Levetiracetam	Partial and generalized epilepsy	The mechanism is unclear. It is suggested that the binding to synaptic vesicle 2A is the key factor in its action	Neuroprotective in PD	Counteracts the effect of pathological mutant expression of LRRK2 G2019S. It is a specific neuroprotectant on the mutant pathological toxicity of LRRK2	Three cell models: Primary cortical neurons obtained from C57BL/6 LRRK2 WT and LRRK2 G2019S BAC mice PC12 cells expressing doxycycline (dox) inducible LRRK2 G2019S mutant SH‐SY5Y cells expressing the dopamine D2 receptor-bearing a Flag epitope	[8]
Semaglutide	Type II diabetes mellitus	It binds selectively to the GLP-1 receptor and stimulates insulin synthesis, causing a decrease in blood glucose	Neuroprotective in PD	Improves motor disturbances, reduces the decrease in TH levels, the accumulation of α-syn, and increases the expression of GDNF that protects dopaminergic neurons in the substantia nigra and the striatum	Mouse model of chronic PD with MPTP Seventy-two male C57BL/6 mice of 8 weeks of age were used	[10]
Vitamin B12	Vitamin B12 deficiencies	Cofactor for the enzyme methionine synthase, essential for synthesizing purines and pyrimidines	Neuroprotective in PD	AdoCbl modulates the activity of LRRK2, which leads to alterations of protein conformation and ATP binding in LRRK2 (inhibits kinase activity)	Mouse model. BAC LRRK2 (R1441G) and BAC LRRK2 (G2019S) transgenic mice, male, 3 to 5 months of age, and their non-transgenic littermates for LRRK2 kinase inhibition in striatal brain slices	[11]
Pomalidomide	Multiple myeloma	Antineoplastic activity, inhibits proliferation and induces apoptosis of various tumor cells	Neuroprotective in PD	TNF-α inhibitory activity. In Drosophila, inhibition of inflammatory pathways triggered by the Eiger ortholog may be the main mechanism	LRRK2^WD40^ model of PD. Drosophila melanogaster, with LRRK2 loss-of-function mutation in the WD40 domain. Adult wild type and LRRKWD40 mutants males were used	[47]
Dabrafenib	Metastatic melanoma with the BRAF V600E mutation	Inhibits B-Raf kinase activity and decreases the proliferation of tumor cells that contain a mutated BRAF gene	Neuroprotective in PD	It inhibits apoptosis and enhances the phosphorylation of ERK. There is a protein–protein interaction between B-Raf and Rit2 (RIT2, PD risk gene)	Cellular model: SH-SY5Y human neuroblastoma cells and HEK293T cells were used Animal model: C57BL/6 J mice, 8 to 12 weeks old, 20 to 25 g, were used	[56]
Ketoconazole	Fungal infections	Interacts with 14-α-sterol demethylase, inhibit the synthesis of ergosterol, increasing the permeability of fungal cells	Neuroprotective in PD	Mechanism not suggested. The increase in dopaminergic neuron death was stopped	Drosophila transgenic model of PD. The UAS-alpha-synuclein transgenic strain was generated using an attp40 insertion site strain and the Drosophila PhiC31 system	[57]
Felodipine	Mild to moderate essential hypertension	Decreases vasoconstriction by inhibiting the entry of calcium ions through voltage-gated L-type calcium channels	Neuroprotective in PD	Eliminates mutant α-syn in the brain of mice	Zebrafish model and murine. The atg7 mutant fish line (atg7sa14768) and two different neurodegenerative disease mouse models (HD-N171–82Q mice and SNCA (A53T) G2-3 mice) and an mRFP-GFP-LC3 reporter line were used	[58]
Raloxifene	Osteoporosis in postmenopausal women	SERM, increases the expression of proteins in the bone matrix	Neuroprotective in PD	It prevents the loss of dopaminergic neurons in the myenteric plexus, avoiding the increase in pro-inflammatory macrophage density	Mouse model of PD with MPTP. Male C57BL/6 mice, ten weeks old, divided into 6 groups of 8 to 9 mice	[59]
Omarigliptin	Type II diabetes mellitus	Inhibitor of DPP-4	Neuroprotective in PD	Increasing GLP-1 and other hormone levels by inhibiting the degrading enzyme DPP-4	Murine model. Twenty-four rats were used, weighing 200 g ± 25, randomly assigned into four groups (n = 6)	[60]
Triflusal	Prophylaxis of thromboembolic disorder	Acetylation of the active group of COX-1 prevents the formation of thromboxane-B2 in platelets	Neuroprotective in PD	It increases endogenous FGF20 production both in the nigrostriatal tract and in the ventral mesencephalic	6-OHDA lesioned rat model. 120 adult male Sprague Dawley rats, 250 to 280 g	[61]
Candesartan	High blood pressure, heart failure	AT1 receptor antagonist. The antihypertensive action is due to the decrease in systemic peripheral resistance	Neuroprotective in PD	AT1 blockers lead to a decrease in the number of OX6-ir microglial cells, expression of CD68 mRNA, NADPH activity, expression of markers of the M1 phenotype, and α-syn-induced dopaminergic neuronal death	α-syn overexpression model, in AAV9-α-syn vector. Adult male Sprague–Dawley rats, 8 to 10 weeks old, n = 220. Subgroup B1 (n = 28) was treated with vehicle, subgroup B2 (n = 24) with candesartan, and subgroup B3 (n = 24) with telmisartan	[62]
Telmisartan	Hypertension	AT1 receptor antagonist. It binds selectively, blocking their effects and decreasing systemic vascular resistance	Neuroprotective in PD	AT1 blockers lead to a decrease in the number of OX6-ir microglial cells, expression of CD68 mRNA, NADPH activity, expression of markers of the M1 phenotype, and α-syn-induced dopaminergic neuronal death	α-syn overexpression model, in AAV9-α-syn vector. Adult male Sprague–Dawley rats, 8 to 10 weeks old, n = 220. Subgroup B1 (n = 28) was treated with vehicle, subgroup B2 (n = 24) with candesartan, and subgroup B3 (n = 24) with telmisartan	[62]
Nitazoxanide	Gastrointestinal infections	Cell membrane injury in parasites and depolarizes the mitochondrial membrane	Neuroprotective in PD	Loss in OCR and ATP production are improved. It confers protection against the loss of TH-positive neurons of the SN	Mouse model of acute PD with MPTP. Male C57BL-6 J mice, 6 to 8 weeks old, 22 to 25 g, in 6 groups of 6 animals	[63]
Metformin	Type II diabetes mellitus	It inhibits the activity of mitochondrial complex I. Lowers blood glucose levels by decreasing gluconeogenesis and decreasing intestinal glucose absorption	Neuroprotective in PD	It rescued TH-positive neurons, restored DA depletion and behavioral disturbances. Neuroprotection could be mediated by inhibition of α-syn phosphorylation and induction of neurotrophic factors Protects rotenone-induced dopaminergic neurodegeneration by reducing lipid peroxidation	Mouse model of subchronic PD with MPTP. Adult male C57BL/6 mice, 10 weeks old, 20 to 25 g, in 4 groups with 6 mice Mouse model of PD with rotenone. C57BL/6 mice were given an injection of saline or rotenone (2.5 mg/kg/day, ip) for 10 days	[64] [65]
Nilotinib	Chronic myelogenous leukemia	It inhibits the tyrosine kinase activity of the BCR-ABL protein (oncogene that causes myelogenous leukemia)	Neuroprotective in PD	Inhibits the enzyme c-Abl. In PD, this protein loses its original shape and forms aggregates that the brain cannot discard and damage neurons	Clinical trial. Single-center, phase 2, randomized, double-blind, placebo-controlled trial with 75 patients randomized 1:1:1 to placebo; nilotinib 150 mg; or nilotinib 300 mg	[9, 50]
Exemestane	Advanced breast cancer in postmenopausal women	It binds irreversibly to the aromatase active site, reduces estrogen concentrations. This delays tumor growth and disease progression	Neuroprotective in PD	It activates the Nrf2 signaling pathway, induces the gene expression of NQO1, HO-1, and GCL, and suppresses inflammatory responses. By elevating antioxidant enzymes, it appears to protect nigral dopaminergic neurons	Cell cultures. BV-2 murine microglial cells and CATH. Murine dopaminergic neuronal cells were cultured Murine model. Male C57BL/6 J mice, 23 to 25 g, 8 weeks old, four groups (n = 10); vehicle-treated; MPTP; MPTP plus 1 mg/kg exemestane; MPTP plus 10 mg/kg exemestane	[67]
Salbutamol	Bronchospasm and other chronic bronchopulmonary disorders	Activation of β2AR in airway smooth muscle leads from cAMP activation to muscle relaxation	Neuroprotective in PD Associated with a lower risk of PD	It increases endogenous FGF20 production in the nigrostriatal tract and can potentially impact the survival of dopaminergic neurons The β2AR ligands modulate the α-syn gene’s transcription (SNCA) through the acetylation of histone 3 lysine 27 from its promoter	6-OHDA lesioned mouse model. 120 adult male Sprague Dawley rats, 250 to 280 g. 80 rats in the in vivo screening and 40 in the neuroprotection study with 6-OHDA The effects of β2AR activation were evaluated in a mouse model of human parkinsonism induced by MPTP and in a neuronal culture system derived from induced pluripotent stem cells	[61] [68]
Pentamidine	*Pneumocystis carinii* pneumonia	The exact mechanism is unclear. It is believed to interfere with nuclear metabolism	Improves motor performance in PD	It produces inhibition of S100B, which inhibits the RAGE/NF-κB pathway in the nigrostriatal circuit, giving an improvement in motor performance	Mouse model of PD with MPTP. Male C57Bl/6 J mice, 8 weeks old	[69]
Ceftriaxone	Bacterial infections (antibiotic)	The beta-lactam fraction binds to carboxypeptidases, endopeptidases, and transpeptidases in the bacterial cytoplasmic membrane; bacteria produce defective cell walls	Anti- LID	Can attenuate the loss of TH together with an increase in glutamate uptake and the expression of the glutamate transporter GLT-1, this increase could reach the threshold of the expression level of GLT- 1 needed to prevent or reduce LID	Rat model of 6-OHDA. Male Sprague Dawley rats (N = 38), 4 to 9 months old. The study was carried out in replicas in the three participating institutions	[12]
Vilazodone	Antidepressant	The exact mechanism is unclear. It is known to selectively inhibit serotonin reuptake and act as a partial agonist at 5HT-1A receptors	Anti- LID	It selectively inhibits L-DOPA-induced gene regulation in the direct pathway of the dopamine-depleted striatum	Hemiparkinsonian rat model injured with 6-OHDA Mice were randomly divided into four experimental groups (n = 8 each). A subacute model of MPTP toxicity induced experimental parkinsonism in mice	[54, 55]
Methylene blue	Acquired methemoglobinemia	It reacts within red blood cells, converts the ferric ion (Fe^3+^) to its oxygen-bearing ferrous state (Fe^2+^)	Anti- LID	Antidyskinetic effects are likely to occur through inhibition of sGC in the CNS	6-OHDA lesioned rat model. Adult male Wistar rats, 200 to 250 g	[70]
Nalbuphine	Analgesic (moderate to severe pain)	The exact mechanism of action is unknown, but it is believed to interact with an opiate receptor site in the CNS	Anti- LID	Striatum analyzes showed that nalbuphine co-therapy blocks several molecular pathways of LID	Model of PD in non-human primates treated with MPTP. Macaques with advanced parkinsonism and reproducible LID received subcutaneous treatment as monotherapy, acute coadministration with levodopa, and chronic coadministration for 1 month	[71]
Ketamine	General anesthetic	It interacts with N-methyl-D-aspartate (NMDA) receptors, opioid receptors, muscarinic, monoaminergic, and voltage-sensitive Ca ion channels	Anti- LID	The effect is mediated by the release of BDNF in the striatum, followed by activation of ERK1 / 2 and mTOR signaling. This leads to a reduction in the mushroom spines’ density, a phenotype highly correlated with LID	LID rodent model Two Sprague–Dawley rats, male, adult, and about 225 g The severity of the LID was evaluated by an investigator blinded to the experimental conditions	[72]
Dimethyl fumarate	Multiple sclerosis	It is not very well known. It is believed to upregulate the Nrf2 pathway that is activated in response to oxidative stress	PD-associated synucleinopathy	Activates NRF2 in the basal ganglia, protects nigral dopaminergic neurons against α-syn toxicity, and decreases astrocytosis and microgliosis	Nrf2 − / − and Nrf2 + / + mice. An adeno-associated pseudotype 6 (rAAV6) viral vector was used to express human α-SYN under the neuron-specific human Synapsin 1 promoter	[73]
Kanamycin	Bacterial infections (antibiotic)	It binds to four nucleotides of the 16S rRNA, which interferes with the initiation complex	PD-associated synucleinopathy	It effectively inhibits the solution phase and lipid-induced aggregation of α-syn	The effect of Kanamycin on the binding affinities of Α-Syn towards both the model and mimic SUVs was studied using a specific lipid-staining fluorescent probe DiIC-18 (DiD)	[74]
Incyclinide o CMT-3	Reduced antibiotic activity	They have been used in trials to treat HIV infection, among others, for which the specific mechanisms are not yet known	PD-associated synucleinopathy	Inhibits α-syn amyloid aggregation. Disassembles α-syn fibrils into smaller fragments that cannot be seeded in subsequent aggregation reactions (fibril extraction mechanism)	Cell cultures in SH-SY5Y. SH-SY5Y cells were incubated with α-synuclein oligomers prepared in the absence or the presence of CMT-3, and an LDH assay measured cytotoxicity	[75]
Doxycycline	Bacterial infections (broad-spectrum antibiotic)	It inhibits translation by binding to the 16S rRNA portion of ribosome 9, preventing the binding of tRNA to the 30S subunit	PD-associated synucleinopathy	It reforms the oligomers of α-syn and inhibits their aggregation, thus avoiding cytotoxicity in dopaminergic cells	Human neuroblastoma cell culture. SH-SY5Y cells were grown in DMEM supplemented with fetal bovine serum	[76]

*AdoCbl* Adocobalamina, *AT1* Angiotensin II type 1, *BDNF* Brain-derived neurotrophic factor, *cAMP* Cyclic adenosine monophosphate, *CMT-3* Tetracycline 3 modified chemically, *CNS* Central nervous system, *COX*-*1* Cyclooxygenase-1, *DA* Dopamine, *DPP*-*4* Dipeptidyl peptidase-4, *ERK* Extracellular Signal–Regulated Kinase, *FGF20* Fibroblast growth factor 20, *GCL* Ganglion cell layer, *GDNF* Glial cell line–derived neurotrophic factor, *GLP*-1 Glucagon-like peptide 1, *HO*-1 Heme oxygenase-1, *LID* L-DOPA-induced dyskinesia, *LRRK2* Leucine-rich repeat kinase 2, *mTOR* Mammalian target of rapamycin, *MPTP* 1-methyl-4-phenyl-1, 2, 3, 6-tetrahydropyridine, *NF*-*kB* Factor nuclear-kappa B, *NQO1* NADPH: quinone oxidoreductase 1, *OCR* Oxygen consumption rate, *PD* Parkinson’s disease, *RAGE* Receptor for advanced glycation end products, *SERM* Selective estrogen receptor modulator, *sGC* Soluble guanylyl cyclase, *TH* Tyrosine hydroxylase, *TNF*-*α* Tumor necrosis factor α, *α*-*syn* α-Synuclein, *6*-*OHDA* 6-Hydroxydopamine

The previous studies demonstrated conclusive evidence in in vitro and in vivo models. In addition to these, a search was carried out on https://clinicaltrials.gov, and six drugs with evidence from clinical trials were found as possible treatments for PD.

*Exenatide* It is one of the most studied drugs and has promising results for successful repositioning. At least until the first half of 2021, three trials were found in the recruitment status, two in an active status, one terminated, and one in an unknown status (a study that has passed its end date and has not had a status update in more than 2 years). The study with the identifier NCT01971242 was concluded in November 2016. Its main objective was to compare exenatide’s effectiveness versus placebo in the motor subscale MDS-UPDRS (Movement Disorder Society‐Unified Parkinson’s Disease Rating Scale) in patients with PD of moderate severity. A phase II study (research phase to describe clinical trials that collect preliminary data on drug efficacy and assess the safety and short-term adverse events) with 60 participants, with a double-blind, placebo-controlled trial. Exenatide had positive effects on motor scores. However, it is unknown whether exenatide affects PD’s pathophysiology or induces long-lasting symptomatic effects. Nevertheless, exenatide represents an encouraging proposal for reuse in PD.

*Nilotinib* We found three clinical trials registered on https://clinicaltrials.gov, one in an unknown state and two in a terminated state for this drug. The most recent completed study with identifier NCT03205488 published its first results in July 2020; it was randomized, double-blind, placebo-controlled, phase II, parallel-group, two cohorts, to define the safety, tolerability, and biological activity of chronic administration of nilotinib in participants with PD. In this study, daily oral administration of nilotinib was evaluated as a chronic treatment of PD symptoms. The results demonstrated acceptable safety and tolerability of nilotinib. However, the low CSF (cerebrospinal fluid) exposure combined with the trend-negative efficacy data led the authors of this clinical trial to suggest that nilotinib should not be further tested in PD. Recently (March 2021), Simuni et al. [66] reported that in a phase II clinical trial, it failed to change levels of dopamine and associated it with the fact that nilotinib has low exposure to cerebrospinal fluid, indicating poor brain penetration; therefore, these assays could be optimized by reformulation in functionalized nanoparticles (NPs). Considering the results of other trials where efficacy data have been reported, we suggest that clinical trials should continue to optimize drug delivery to the Central nervous system (CNS).

*Levetiracetam (LEV)* This drug currently presents three clinical trials in the terminated phase, one in the suspended phase, one in the recruiting phase, and one in the unknown phase. The three completed trials are on the anti-LID activity of LEV in PD, and no conclusive results have been published. A phase IV trial, with identifier NCT00307450, was concluded in July 2009, conducted as a randomized, double-blind, placebo-controlled, parallel-group pilot study in PD patients with moderate to severe LID on stable dopaminergic treatment. This study aimed to evaluate the efficacy, safety, and tolerability of LEV for the treatment of LID in PD, and it was observed that LEV had mild antidyskinetic effects without worsening parkinsonian symptoms or compromising the efficacy of levodopa.

*B12 vitamin* A clinical trial of vitamin B12 for PD has been found. This study with the ClinicalTrials.gov identifier: NCT00208611 was a phase III trial, and its objective was to evaluate the status of cobalamin and the response to supplementation in patients with PD. However, this trial was unsuccessful and terminated because funding ended, and patient enrollment was not completed within the specified time frame. This study could also provide critical pilot data to evaluate treatment efficacy for patients considered to have below-normal serum vitamin B12 levels.

*Ceftriaxone* This drug is currently in a clinical trial under the ClinicalTrials.gov identifier: NCT03413384. It is in recruitment status and is a phase II trial, with an estimated study completion date of May 2022. This study evaluates the efficacy and safety of ceftriaxone in patients with mild PD dementia. The effects observed in the animal model of PDD (Parkinson’s disease dementia) have suggested that ceftriaxone is a potentially promising medical treatment for PDD patients to improve cognitive and motor function defects.

*Semaglutide* This drug is one of those recently proposed for reuse in PD that has reached clinical trials; it is currently in a trial with the ClinicalTrials.gov identifier: NCT03659682. This study is in a state of “not yet recruiting,” with an estimated completion date of December 2024 to test the neuroprotective and anti-inflammatory properties of semaglutide in PD.

These clinical trials expose us to how promising drug repositioning could be in PD. There are drugs with satisfactory results to suggest more comprehensive studies, such as exenatide, levetiracetam, and nilotinib, on the right path to repositioning. On the other hand, some trials have remained in an “unknown” state, which has not been given continuity and for which it is necessary to carry out a more detailed review of the possible causes of not reporting the findings.

## Pharmaceutical nanotechnology strategies for optimizing drug repositioning in Parkinson’s disease

Pharmaceutical nanotechnology enables novel approaches to drug delivery [77]. One promising approach is NPs as carriers for drug transport. NPs allow overcoming pharmacological limitations such as low solubility, rapid biodegradation, low bioavailability, adverse effects, and low permeability through biological barriers [78]. The main challenges in developing a formulation to treat PD include an efficient crossing of the drug through the BBB and controlled drug release to avoid fluctuations in concentration. The BBB has the function of protecting the CNS, restricting the entry of harmful xenobiotics, regulating the passage of endogenous molecules, and limiting therapeutic agents’ entry [79, 80]. Because PD therapies are generally chronic, taking medications by mouth is a comfortable and safe option for patients; however, studies of NPs with the ability to overcome both the BBB and the gastrointestinal barrier are needed. Currently, the approaches for administering drugs through the BBB are direct injection and implantation, the temporary opening of the BBB, intravenously (IV), and intranasally [81–86]. An effective nanocarrier of drugs for PD should ideally be capable of protecting the drug from physiological conditions, overcoming the BBB and target neurons in the brain, and guaranteeing a controlled release at the site of action [19, 87]. Several internalization mechanisms have been found through the BBB that are mainly influenced by the surface properties of NPs; these are receptor-mediated endocytosis, adsorption-mediated endocytosis, macropinocytosis, and opening of tight junctions [88–91].

### Biodegradable nanoparticles

**Fig. 3:**
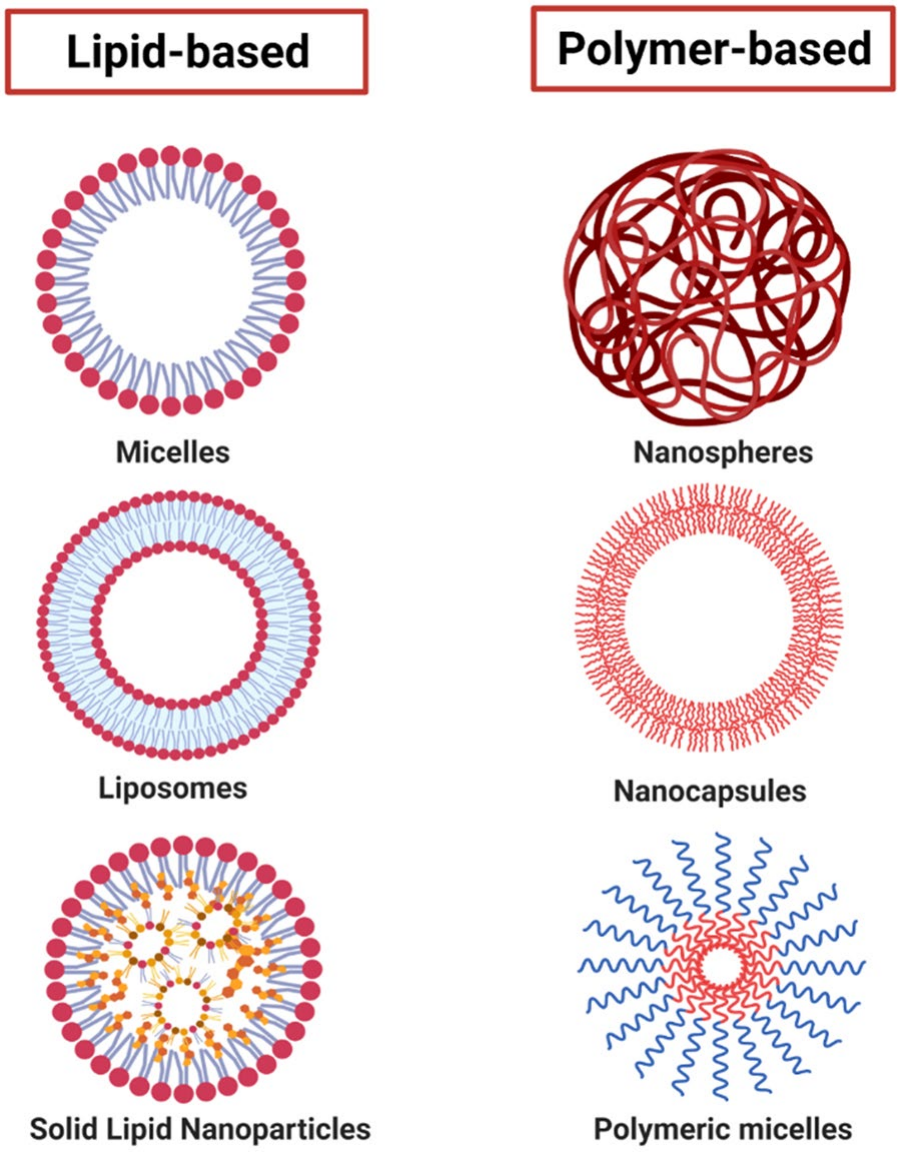
Biodegradable nanoparticles. Nanosystems are proposed for the optimization of drug delivery in PD. NPs based on lipids and polymers are the most interesting since they are synthesized based on biodegradable and biocompatible materials representing low toxicity and a high capacity to modify their physicochemical properties

NPs can be synthesized from different materials offering variable physicochemical characteristics, which allow different interactions with biological systems. We suggest using biodegradable NPs (polymeric and lipid, Fig. 3) in drug reformulation for optimal repositioning in PD since they offer numerous advantages over other materials (for example, metallic or ceramic). Currently, there is plentiful research on synthesis procedures and applications of polymeric NPs, the use of biopolymers has the advantage that there are well-established methods for their development, and there is extensive information on their toxicity. Also, polymers offer a high capacity to modify their physicochemical properties to synthesize NPs [19]. Some of the most successful polymeric materials in their use are gelatin, hyaluronic acid, alginate (ALG), chitosan (CS), polylactic-co-glycolic acid (PLGA), polylactide (PLA), polyethylene glycol (PEG), polycaprolactone (PCL), and polyanionic cellulose (PAC) [78]. Lipid nanoparticles have certain advantages that make them attractive to be used as nanocarriers in PD [92], mainly their composition of lipid matrix (based on phospholipids, cholesterol, triglycerides) that is physiologically tolerable, leads to little toxicity, scalability of production without the need for organic solvents, and their high bioavailability [93].

#### Physicochemical parameters for the optimization of NPs

There is currently no consensus on the physicochemical characteristics that NPs must meet to achieve greater drug delivery efficiency to the CNS since these characteristics depend on the specific materials used in their synthesis. Relatively large-sized NPs, but with suitable surface coatings (e.g., non-ionic surfactants, cationic polymers), have been found to pass through the BBB; therefore, it is advisable to evaluate each proposed nanoformulation in vitro and in vivo. However, for the design of NPs targeting the CNS, certain parameters must be considered in a general way, such as size, PDI, morphology, drug load, and Z potential.

Concerning size, it has been reported that there is greater absorption as size decreases; NPs of 100 nm in diameter are significantly more absorbed than larger particles [94, 95]. Another study showed that the smallest NPs, between 50 and 100 nm, do not exhibit a significant difference in cell absorption [96]. On the other hand, it has been documented that even NPs of 345 nm [19] and up to 422 nm can cross the BBB (these larger-sized NPs were functionalized with non-ionic surfactants) [97]. In 2020, Lombardo et al. conducted an extensive review, gathering data from more than 50 articles reporting NPs with sizes between 100 and 345 nm with efficient crossing through the BBB [98]. Gao and Jiang studied the influence of particle size on the transport of methotrexate through the BBB by polysorbate 80-coated polybutylcyanoacrylate NPs. They studied NPs with sizes from 70 to 345 nm, finding that NPs between 170 and 345 nm did not present a significant difference in methotrexate delivery to the brain [99]. Therefore, we suggest that a size of NPs between 100 and 345 nm could be used as a reference point to start testing for BBB internalization. The PDI must be less than 0.1 for a monodisperse size distribution to be considered [100].

Concerning morphology, spherical NPs are preferred because they guarantee an adequate volume/contact surface ratio [101]. Regarding drug loading, it is preferable to transport drugs in NPs with high loading capacity to ensure greater delivery of drugs with a low number of NPs and to avoid the toxic accumulation of materials used to synthesize NPs [19]. The drug loading capacity in NPs is a difficult parameter to control; most of them have the drawback of low drug loading (generally less than 10%); therefore, nanosystems with high drug loading capacity are necessary, which reach a drug load greater than 10% [102]. Compared to the physical encapsulation of drug molecules in inert carriers, polymer-drug conjugates are good candidates for NPs with high drug loading due to their limited use of carrier materials. In this context, Shen et al. developed linear conjugates of polymer-drug by conjugating one or two molecules of strong hydrophobic camptothecin (CPT) to a very short oligoethylene glycol chain, reaching a drug load content of 40 to 58% [103]. For brain-targeted drugs that need to cross the blood–brain barrier, carrier materials are essential for their function, and some materials allow good drug loading. For example, PLGA NPs with a size of ~ 184. 6 nm have achieved a drug loading capacity of 10.21 ± 0.89% [104]. Other PLGA NPs with a size of ~ 155 nm reached their highest drug loading capacity of 20.6% [105]. The influence of the Z potential, on the one hand, allows controlling the stability of NPs in solution; on the other hand, it allows controlling the interaction with the biological environment. For greater stability of NPs only by electrostatic repulsion, an absolute minimum Z potential value of |30 mV| is required, approximately |20 mV| provides short-term stability, and values in the range of |5 mV| indicate low stability (rapid aggregation) [106]. In the case of a stabilization of NPs that combine electrostatic repulsion and steric stabilization (electrosteric stabilization), generally, it is required to have a minimum value of |20 mV|. The NPs are stabilized with the help of non-ionic surfactants; therefore, the resulting steric effect contributes substantially to the stability of NPs with zeta potentials below |20 mV| [106, 107]. Although, due to the negative surface charge of the endothelial cells of the BBB, NPs with positive Z potential are preferable to promote bioadhesion (by the principle of electro-attraction) and, consequently, the permeability of the BBB.

Upon contact with biological matrices, most materials are immediately coated with proteins, forming a protein crown [108]. The affinity for proteins is higher towards hydrophobic nanomaterials or charged surfaces than hydrophilic or neutral ones [109]. Neutrally charged NPs have been shown to have a distinctly slower opsonization rate than charged particles [110]. A study on the influence of the zeta potential of negatively charged polymeric NPs showed an increase in plasma protein uptake with increasing surface charge density [111]. Nanomaterials with hydrophobic surfaces have an affinity for adsorbing apolipoproteins, albumin, and fibrinogen, whereas hydrophilic surfaces bind a smaller proportion of these proteins [112]. Therefore, the formation of the protein corona cannot be completely avoided. An option is to adhere to materials with a nearly neutral charge or highly hydrophilic on the surface of the NPs; if the adhesion of proteins is completely avoided, NPs could become toxic [19].

#### Surface functionalization

**Fig. 4:**
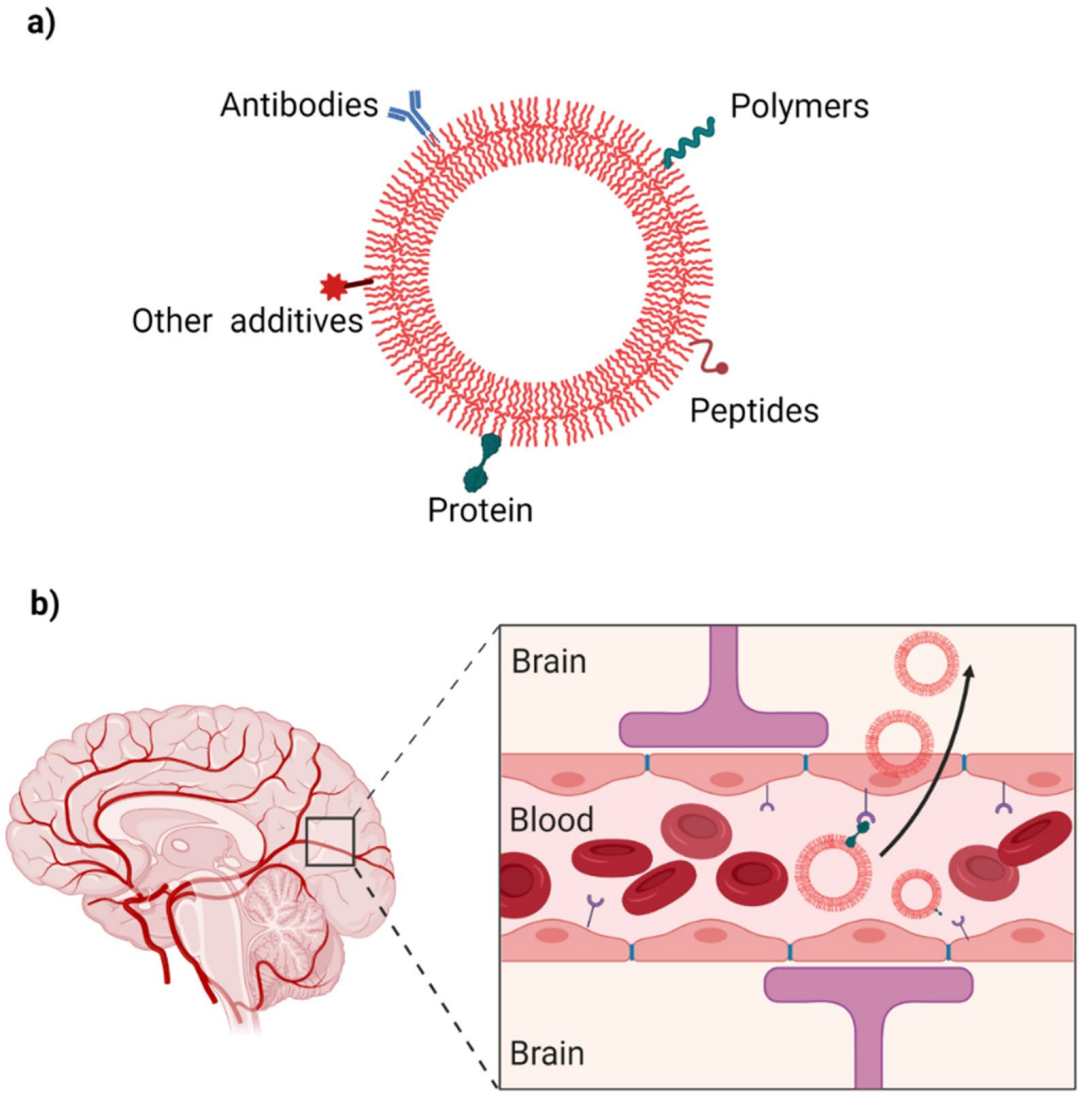
**a** Increased specificity towards BBB. The surface coating of NPs with suitable materials can increase the specificity towards the BBB; these materials (as mentioned in Table 2) can be polymers, proteins, antibodies, peptides, and other additives. **b** Receptor-mediated transcytosis. The passage through the BBB is exemplified through receptor-mediated transcytosis. A very frequently used pathway for the transport of NPs to the brain, for this use, is assembled with specific receptors found in the BBB

NPs surface functionalization allows materials to be added to the surface layer to target NPs to specific receptors found on particular cell types (e.g., dopaminergic neurons) and improve cell permeability. For this reason, various materials such as polymers, proteins, and other additives have been assessed [113] (Fig. 4a). In Table 2, we cite examples of potential materials used as NPs surface linkers for drug delivery in PD. A study has suggested and successfully demonstrated that membrane factors, such as transferrin receptors (TfRs), can promote NPs transcytosis by specific interaction with gastrointestinal endothelial cells [114]. At the brain level, lactoferrin (Lf) is a ligand that favors the absorption of NPs in the BBB since there is an increase in the expression of lactoferrin receptors (LfRs) in the substantia nigra and striatal dopaminergic neurons, as well as in the endothelial cells of the BBB of PD patients [115, 116]. Thus, the efficacy of NPs in PD can be improved by functionalizing the surface with Lf and Tf that act as ligands to promote receptor-mediated transcytosis (Fig. 4b).

**Table 2:** Examples of materials to functionalize the surface of NPs proposed for drug delivery in PD

Composition of NPs	Functional material	Function	Mechanism	References
7pep-M-C6	Transferrin	Crossing of gastrointestinal barrier	Enter the cells through a specific clathrin-mediated mechanism	[114]
DA-Tf-LP	Transferrin	Crossing of BBB	BBB crossing by Tf receptor-mediated endogenous transcytosis	[117]
B-Lf-PEG-PLGA	Lactoferrin	Effective biological ligand to the striatum	The Lf receptor is overexpressed in epithelial cells, capillaries, and neurons in PD. Cellular uptake occurs via receptor-mediated transcytosis to Lf	[115]
DA-PEG-LP	Polyethylene glycol	Evasion of the immune system	PEG coating is believed to increase its biological half-life due to reduced interactions with plasma proteins or cell surface receptors	[118]
Selegiline-CS	Chitosan	Crossing of BBB and mucosal barriers	The mucoadhesive nature of QS improves mucosal retention time, improves permeability through the BBB through endocytosis by electrostatic adhesion, and through an opening of tight junctions	[119]
pDNA-NGF-GNP	Nerve growth factor	Improves neural uptake	Enhances neuronal uptake through NGF receptor-mediated endocytosis	[120]
hGDNF-Angiopep-DGL-PEG	Angiopep	Crossing of BBB	Angiopep is a ligand that specifically binds to low-density lipoprotein receptor-related protein (LRP that is overexpressed on the BBB and crosses by transcytosis	[121]
RHCl-Polysorbate 80-CS	Polysorbate 80	Crossing of BBB	Coating with polysorbate 80 helps in the adsorption of plasma proteins from blood and thus, facilitates the entry of nanoparticles to BBB by the receptor-mediated endocytosis	[122]

*B* Borneol, *CS* Chitosan, *C6* Coumarin 6, *DA* Dopamine, *DGL* Dendrigraft poly-L-lysine, *GNP* Gold nanoparticles, *hGDNF* Human glial-derived neurotrophic factor, *Lf* Lactoferrin, *LP* Liposomes, *NGF* Nerve growth factor, *NPs* Nanoparticles, *M* PEG-b-PCL copolymer, *pDNA* Plasmid DNA, *PEG* Polyethylene glycol, *PLGA* Poly Lactic-co-Glycolic Acid, *RHCl* Ropinirole hydrochloride, *Tf* Transferrin, *7pep* Transferrin receptor specific 7peptide

### Current advances in NPs for Parkinson’s disease

Reports detailed the presentation of drugs currently being used for PD and have been reformulated in NPs. For instance, Zhao et al. [123] developed polymeric NPs based on PEG–PCL. The formulation encapsulated Ginkgolide B (GB), which is believed to act in a neuroprotective way and treat PD. GB has poor oral bioavailability, limiting its clinical application, and these NPs facilitated sustained release, thus enhancing its ability to accumulate in the brain and treat PD. The NPs had a size of 91.26 ± 1.34 nm, polydispersity index (PDI) of 0.17 ± 0.01, the zeta potential of − 12.09 ± 0.97 mV, load capacity of 26.93%, and encapsulation efficiency (EE) of 87.52%. A biphasic release pattern was observed, and ~ 30% of the total GB was released during the first two h, followed by a more gradual sustained release of 94% until a 48 h period. This characteristic could be improved by playing with polymer concentrations or even coating the surface with other polymers such as CS. Bromocriptine (BRC) is a widely used PD drug that slows down and minimizes the motor fluctuations associated with L-DOPA. Shadab et al. [124] developed BRC-loaded CS NPs with an average size of 161.3 nm, a zeta potential of 40.3 mV, load capacity 37.8%, EE of 84.2%, and increased brain activity uptake of BRC-NPs was observed. Gambaryan et al. [125] developed PLGA NPs loaded with L-DOPA, with a size of 250 ± 50 nm and EE of 10 ± 2%. The authors recorded an L-DOPA-PLGA-NPs increased motor function during the treatment period of 112 days by the intranasal route, demonstrating a prolonged effect even one week after the interruption of treatment with the possible reduction of the effective drug dose and the frequency of administration. Fernandes et al. [126] developed PLGA-PEG NPs, as carriers of coumarin, a potent drug inhibitor of monoamine oxidase B (MAO-B), reversible and selective, but with suboptimal aqueous solubility, which prevents its use in vivo tests. The NPs had an average size of 105 nm, a zeta potential of − 10.1 mV, and EE of 50%. The PLGA NPs inhibited P-glycoprotein (P-gp) and could cross the intestinal and brain membranes, allowing the successful transport of coumarin to the brain. In these reports, polymeric materials (CS, PCL, PEG, and PLGA) are attributed to the ability to have an affinity for the BBB and to be able to permeate it effectively.

Reports of functionalized nanocarriers have also been found, which have presented promising results for PD use. Lopalco et al. [117] developed liposomes (LP) loaded with dopamine hydrochloride (DA HCl) functionalized with Tf, with a size of 181.7 ± 7.8 nm, EE of 35.4 ± 1.8%, PDI of 0.2, and potential zeta of + 7.5 mV. Stability was evaluated by measuring their size and PDI for one month; then, the amount of DA was determined by high-performance liquid chromatography (HPLC), and no significant variations were detected, so it was stated that the vesicles are stable and can be used for future studies. With these LP, an improvement of the crossing of the BBB was achieved, increasing the benefits and reducing the complications of patients undergoing chronic treatment with L-Dopa. On the other hand, Huang et al. [127] developed polyamidoamine (PAMAM) NPs and PEG functionalized in the same way with Lf, with an average size of 196 nm, a zeta potential of 29.35 mV, and loaded with plasmids for neurotrophic factor derived from the human glial cell line (hGDNF). GDNF is considered the gold standard neurotrophic factor for PD therapy. However, GDNF cannot cross the BBB; thus, the formulation in NPs becomes capable of crossing the BBB and exerting a neuroprotective effect on dopaminergic neurons.

### Reformulation strategies of NPs for Parkinson's disease

In the present review, we have identified the drugs currently being proposed for drug repositioning and the areas of opportunity for a possible reformulation in NPs (Table 3) that allow future repositioning studies to be optimized. Their ability to cross the BBB was also identified, and whether they have been previously reformulated into NPs.

**Table 3:** Drugs with areas of opportunity for reformulation in NPs for PD

Drug	Clinical trial status	Cross the BBB?	Formulated in NPs? (Type/composition)	Area of opportunity
Exenatide	3–Recruiting 2–Active 1–Terminated 1–Unknown	Yes	Polymeric NPs/CSK-DEX-PLGA [128]	Rapidly eliminated by glomerular filtration, reformulation in NPs could increase its half-life in plasma and avoid enzymatic degradation
Levetiracetam	3–Terminated 1–Suspended 1–Recruiting 1–Unknown	Yes	Polymeric NPs/PLGA [129]	Reformulation in NPs could reduce the dose and administration frequency, reducing side effects
Semaglutide	1–Not yet recruiting	No	Liposome/Phospholipid- cholesterol [130]	NPs could improve stability, bioavailability, and passage through the BBB and avoid toxic accumulation due to its half-life of approximately one week
Vitamin B12	1–Terminated	Yes	Lipid-protein NPs/Barley protein-α-tocopherol-Phospholipids [131]	Reformulate in NPs with surface functionalization allows their targeting to the brain
Pomalidomide	N/I	Restricted (P-gp substrate)	N/I	BCS class IV, reformulation in NPs could improve intestinal absorption and permeability through the BBB
Dabrafenib	N/I	Restricted (P-gp substrate)	N/I	Reformulation in NPs with anti-P-gp surface functionalization could allow passage through the BBB
Ketoconazole	N/I	Restricted (P-gp substrate)	Polymeric NPs/PLGA [132]	Low solubility. Reformulation in NPs could offer a controlled release, reduce toxic effects, and achieve greater bioavailability
Felodipine	N/I	Yes	Polymeric NPs/PLGA [133] SLN/Glyceryl behenate [134]	Variable bioavailability, poor solubility, and extensive liver metabolism. Reformulation in NPs could offer greater brain bioavailability
Raloxifene	N/I	Yes	Polymeric NPs/CS [135] SLN/Glyceryl behenate [136]	Low oral bioavailability, poor solubility, and extensive metabolism in the intestine (> 90%). Reformulation in NPs could improve oral bioavailability
Candesartan	N/I	Yes	SLN/Trimyristin-Tripalmitin-Tristearin [137]	Low oral bioavailability, poor solubility. Reformulation in NPs could improve oral bioavailability and target the brain
Telmisartan	N/I	Yes (dose-dependent)	Polymeric NPs/PLA [138]	Low oral bioavailability, poor solubility. NPs could allow greater penetration of the BBB and target the brain
Nitazoxanide	N/I	Low permeability	SLN/Hydrogenated palm oil- Hydrogenated soybean lecithin [139]	Reformulation in NPs could allow more passage through the BBB, greater control of the dosage, and avoid toxic effects
Metformin	N/I	Yes	Polymeric NPs/Alginate [140]	BCS class III, low absorption. Reformulation in NPs could facilitate absorption and control the dosage and release at the specific site of action
Nilotinib	1 – Active 2 – Terminated	Low permeability	Polymeric micelles/Styrene-co-maleic acid [141]	Low exposure to CSF limits its use in PD. Reformulation in functionalized NPs could allow vectorization towards the CNS
Exemestane	N/I	Yes	Polymeric NPs/Alginate [142]	Reformulating in NPs could improve solubility and bioavailability, control release, and decrease side effects
Salbutamol	N/I	Yes	Polymeric NPs/PLGA, and poly(vinyl sulfonate-co-vinyl alcohol)-graft-PLGA [143]	Low oral bioavailability. Reformulation in NPs could allow the targeting of the target neurons
Pentamidine	N/I	Low permeability	Polymeric NPs/PLGA [144] Liposome/Phosphatidylcholine Polymeric NPs/PCL [145]	It can cause diabetes and other toxic effects. Reformulation in NPs could improve permeation through the BBB, greater control of the dosage, and avoiding toxic effects
Ceftriaxone	1 – Recruiting	Yes	Polymeric NPs/CS [146]	It is administered parenterally. Only 1% oral bioavailability, reformulation in NPs could increase its bioavailability and allow a controlled release
Vilazodone	N/I	Restricted (P-gp substrate)	Polymeric NPs/Copolymer Soluplus^®^-Polyvinylpyrrolidone [147]	Low solubility. Reformulation in NPs could increase bioavailability and permeation through the BBB
Methylene blue	N/I	Yes	Metallic NPs/Ag [148]	Rapid distribution in tissues. Severe toxicity in high doses. Reformulation in NPs could allow controlled dosage and vectorization towards the CNS
Nalbuphine	N/I	Yes	SLN/Phosphatidylcholine [149]	They are limited to parenteral use. High concentrations can cause sedation. Reformulation of NPs could allow oral administration and greater dosage control
Ketamine	N/I	Yes	Polymeric NPs/PEG-PLGA [150]	Short half-life. Serious adverse effects. Reformulation in NPs could increase their bioavailability and specific release in target neurons
Dimethyl fumarate	N/I	Low permeability	SLN/Tocopherol acetate [151]	Short half-life. Reformulation in NPs could improve bioavailability, brain permeability and reduce adverse effects
Kanamycin	N/I	Low permeability	Metallic NPs/Au [152]	Relatively insoluble in lipids. Reformulation in NPs could allow greater oral bioavailability and permeation through the BBB
CMT-3	N/I	Yes	N/I	Multi-target drug. Reformulation in NPs could allow targeting of target neurons
Doxycycline	N/I	Yes	Polymeric NPs/PLGA-PCL (153)	Reformulation in NPs would allow the sustained administration of the drug, minimizing adverse effects

*Ag* Silver, *Au* Gold, *BBB* Blood–brain barrier, *BCS* Biopharmaceutical classification system, *CMT-3* Tetracycline 3 modified chemically, *CNS* Central nervous system, *CS* Chitosan, *CSF* Cerebrospinal fluid, *CSK* CSKSSDYQC peptide, *DEX* Dextran, *N/I* No information, *NPs* Nanoparticles, *PCL* Poly ɛ‐caprolactone, *PD* Parkinson’s disease, *PEG* Polyethylene glycol, *P-gp* P-glycoprotein, *PLA* Polylactic acid, *PLGA* Poly lactic-co-glycolic acid, *SiO2* Silicon dioxide, *SLN* Solid lipid nanoparticles

We found that most of the drugs proposed for repositioning in PD have been reformulated in NPs, at least for research purposes. However, most have been reformulated to overcome the gastrointestinal barrier. Therefore, we identified that it is necessary to test these drugs in NPs to improve the BBB crossing and brain bioavailability. The following is evidence of drugs that have been reformulated in NPs, focusing on overcoming BBB; however, not all formulations have had the same effects on the central nervous system. Kumar et al. [140] encapsulated Dimethyl fumarate in solid lipid nanoparticles (SLN) synthesized from tocopherol acetate, with a mean size of 69.70 nm, PDI of 0.317, the zeta potential of − 9.71 mV, EE of 90.12%, and load capacity of 20.13%. The research confirmed higher intestinal absorption and neuronal uptake through cell uptake studies in Caco-2 and SH-SY5Y monolayers, and oral bioavailability increased 4.09 times. Brain bioavailability substantially improved compared to the drug alone. Recently, Khanna et al. [149], encapsulated Nalbuphine (NLB) in SLN synthesized from phosphatidylcholine, with an average size of 170.07 nm, encapsulation efficiency of 93.6%, and loading capacity of 26.67%. NLB-SLN brain targeting was confirmed by noninvasive scintigraphy, reaching its maximum permeability eighth h after intranasal administration. Omarch et al. [145] conducted a comparative study; the authors developed polymeric PCL NPs and phosphatidylcholine LP to evaluate pentamidine in vitro transport through the BBB. The pentamidine-loaded PCL NPs had a mean size of 267.58 nm, PDI of 0.25, and zeta potential of – 28.1 mV, while pentamidine-loaded LP had a mean size of 119.61 nm, PDI of 0.25, and zeta potential 11.78 mV. Pentamidine loading was 0.16 µg/mg (w/w) and 0.17 µg/mg (w/w) in PCL NPs and LP, respectively. LP carried 87% of the dose, PCL NPs 66% of the dose, and free pentamidine penetration was 63% of the dose. Therefore, the results suggested that LP are more efficient nanocarriers for transporting pentamidine through the BBB, at least in vitro. LP were synthesized from L-phosphatidylcholine and cholesterol and, therefore, are considered biocompatible, biodegradable, and less toxic; these reports offer better brain bioavailability of drugs that can be exploited in the better management of PD.

Therefore, NPs are an attractive strategy in the repositioning of drugs for the treatment of PD that can guarantee an adequate therapeutic treatment as an adjuvant or as the main treatment, with a shorter investigation time.

## Conclusion

In PD, drug delivery to affected areas of the brain is desired; however, most molecules cannot cross the BBB, leading to the failure of clinical trials of many drugs proposed for reuse in PD. Drug repositioning in PD is a topic of growing interest to the scientific community and the pharmaceutical industry, as it reduces the number of steps required for clinical development and reduces the amount of time and costs to bring a drug to regulatory approval. Another advantage of repositioning is that the clinical profile of approved drugs is already well characterized. Thus, researchers can often move directly to Phase II evaluations for efficacy trials in the new indication of interest. In this review, we identified 28 drugs that have been proposed as candidates for repositioning in PD in recent years; most of them have an inability or low ability to cross the BBB. To overcome this limitation and optimize future PD repositioning studies, we propose using lipid and polymeric nanosystems: lipid-based NP (SLN, micelles and LP) and polymeric-based NP (nanocapsules, nanospheres and polymeric micelles). These nanosystems show promise for overcoming the pharmacokinetic limitations of conventional therapies. Among their main advantages, they can protect the drug from degradation, provide sustained release, facilitate entry into the CNS, and deliver the drug to specific cells to target particular intracellular pathways. Surface functionalization with polymers, peptides, antibodies, and surfactants, among other materials, is also proposed as a strategy that has been shown to promote efficient crossing through the BBB. Six drugs were found in repositioning clinical trials for PD, of which nilotinib has shown promising results. The current COVID-19 pandemic has evidenced drug repositioning as a hopeful strategy for drug development for difficult-to-treat diseases such as PD, although it has also been evidenced that better protocols and regulations are needed to direct this activity.

## Data Availability

Yes.

## References

[1] GBD 2015 Neurological Disorders Collaborator Group (2017). Global, regional, and national burden of neurological disorders during 1990–2015: a systematic analysis for the Global Burden of Disease Study 2015. Lancet Neurol.

[2] Saavedra Moreno JS, Millán PA, Buriticá Henao OF (2019). Introducción, epidemiología y diagnóstico de la enfermedad de Parkinson. Acta Neurol Colomb..

[3] GBD 2016 Neurological Disorders Collaborator Group (2018). Global, regional, and national burden of Parkinson’s disease, 1990–2016: a systematic analysis for the Global Burden of Disease Study 2016. Lancet Neurol..

[4] Tysnes OB, Storstein A (2017). Epidemiology of Parkinson’s disease. J Neural Transm.

[5] Chen X, Gumina G, Virga KG (2018). Recent Advances in drug repurposing for Parkinson’s disease. Curr Med Chem.

[6] Parisi D, Adasme MF, Sveshnikova A, Bolz SN, Moreau Y, Schroeder M (2020). Drug repositioning or target repositioning: a structural perspective of drug-target-indication relationship for available repurposed drugs. Comput Struct Biotechnol J.

[7] Athauda D, Maclagan K, Skene SS, Bajwa-Joseph M, Letchford D, Chowdhury K (2017). Exenatide once weekly versus placebo in Parkinson’s disease: a randomised, double-blind, placebo-controlled trial. Lancet.

[8] Rassu M, Biosa A, Galioto M, Fais M, Sini P, Greggio E (2019). Levetiracetam treatment ameliorates LRRK2 pathological mutant phenotype. J Cell Mol Med.

[9] Pagan FL, Hebron ML, Wilmarth B, Torres-Yaghi Y, Lawler A, Mundel EE (2020). Nilotinib effects on safety, tolerability, and potential biomarkers in Parkinson disease: a phase 2 randomized clinical trial. JAMA Neurol.

[10] Zhang L, Zhang L, Li L, Hölscher C (2019). Semaglutide is neuroprotective and reduces α-synuclein levels in the chronic MPTP mouse model of Parkinson’s disease. J Parkinsons Dis.

[11] Schaffner A, Li X, Gomez-Llorente Y, Leandrou E, Memou A, Clemente N (2019). Vitamin B 12 modulates Parkinson’s disease LRRK2 kinase activity through allosteric regulation and confers neuroprotection. Cell Res.

[12] Chotibut T, Meadows S, Kasanga EA, McInnis T, Cantu MA, Bishop C (2017). Ceftriaxone reduces L-dopa–induced dyskinesia severity in 6-hydroxydopamine parkinson’s disease model. Mov Disord.

[13] Baskin J, Jeon JE, Lewis SJG (2021). Nanoparticles for drug delivery in Parkinson’s disease. J Neurol.

[14] National Institute of Neurological Disorders and Stroke. Parkinson’s Disease: Hope Through Research. Bethesda, Maryland; 2020. https://www.ninds.nih.gov/Disorders/Patient-Caregiver-Education/Hope-Through-Research/Parkinsons-Disease-Hope-Through-Research. Accessed 21 Dec 2020.

[15] Tanner CM, Kame F, Ross GW, Hoppin JA, Goldman SM, Korell M (2011). Rotenone, paraquat, and Parkinson’s disease. Environ Health Perspect.

[16] Nandipati S, Litvan I (2016). Environmental exposures and Parkinson’s disease. Int J Environ Res Public Health.

[17] Benazzouz A, Mamad O, Abedi P, Bouali-Benazzouz R, Chetrit J (2014). Involvement of dopamine loss in extrastriatal basal ganglia nuclei in the pathophysiology of Parkinson´s disease. Front Aging Neurosci.

[18] Martínez Fernández R, Gasca Salas C, Sánchez Ferro Á, Obeso JÁ (2016). Actualización en la enfermedad de Parkinson. Rev Med Clin Condes.

[19] Leyva-Gómez G, Cortés H, Magaña JJ, Leyva-García N, Quintanar-Guerrero D, Florán B (2015). Nanoparticle technology for treatment of Parkinson’s disease: the role of surface phenomena in reaching the brain. Drug Discov Today.

[20] Zeng XS, Geng WS, Jia JJ, Chen L, Zhang PP (2018). Cellular and molecular basis of neurodegeneration in Parkinson disease. Front Aging Neurosci..

[21] Chen Z, Li G, Liu J (2020). Autonomic dysfunction in Parkinson’s disease: implications for pathophysiology, diagnosis, and treatment. Neurobiol Dis.

[22] Simon DK, Tanner CM, Brundin P (2020). Parkinson Disease epidemiology, pathology, genetics and pathophysiology. Clin Geriatr Med.

[23] Brahmachari S, Karuppagounder SS, Ge P, Lee S, Dawson VL, Dawson TM (2017). c-Abl and Parkinson’s disease: mechanisms and therapeutic potential. J Parkinsons Dis.

[24] Karuppagounder SS, Brahmachari S, Lee Y, Dawson VL, Dawson TM, Ko HS (2014). The c-Abl inhibitor, Nilotinib, protects dopaminergic neurons in a preclinical animal model of Parkinson’s disease. Sci Rep.

[25] Abushouk AI, Negida A, Elshenawy RA, Zein H, Hammad AM, Menshawy A (2017). C-Abl Inhibition; a novel therapeutic target for parkinson’s disease. CNS Neurol Disord Drug Targets.

[26] Martinez-Martin P, Rodriguez-Blazquez C, Forjaz MJ (2012). Quality of life and burden in caregivers for patients with Parkinson’s disease: concepts, assessment and related factors. Expert Rev Pharmacoeconomics Outcomes Res.

[27] Yang W, Hamilton JL, Kopil C, Beck JC, Tanner CM, Albin RL (2020). Current and projected future economic burden of Parkinson’s disease in the U.S. NPJ Park Dis..

[28] Jankovic J, Tan EK (2020). Parkinson’s disease: etiopathogenesis and treatment. J Neurol Neurosurg Psychiatry.

[29] Marsot A, Guilhaumou R, Azulay JP, Blin O (2017). Levodopa in Parkinson’s disease: a review of population pharmacokinetics/pharmacodynamics analysis. J Pharm Pharm Sci.

[30] Schapira AHV, Fox SH, Hauser RA, Jankovic J, Jost WH, Kenney C (2017). Assessment of safety and efficacy of safinamide as a levodopa adjunct in patients with Parkinson disease and motor fluctuations a randomized clinical trial. JAMA Neurol.

[31] Latt MD, Lewis S, Zekry O, Fung VSC (2019). Factors to consider in the selection of dopamine agonists for older persons with Parkinson’s disease. Drugs Aging.

[32] Torti M, Vacca L, Stocchi F (2018). Istradefylline for the treatment of Parkinson’s disease: is it a promising strategy?. Expert Opin Pharmacother.

[33] Cummings J, Isaacson S, Mills R, Williams H, Chi-Burris K, Corbett A (2014). Pimavanserin for patients with Parkinson’s disease psychosis: a randomised, placebo-controlled phase 3 trial. Lancet.

[34] Politi C, Ciccacci C, Novelli G, Borgiani P (2018). Genetics and treatment response in Parkinson’s disease: an update on pharmacogenetic studies. NeuroMolecular Med.

[35] Alonso-Navarro H, Jimenez-Jimenez F, Garcia-Martin E, Agundez J (2014). Genomic and pharmacogenomic biomarkers of Parkinson’s disease. Curr Drug Metab.

[36] Jiménez-Jiménez FJ, Alonso-Navarro H, García-Martín E, Agúndez JAG (2016). Advances in understanding genomic markers and pharmacogenetics of Parkinsons disease. Expert Opin Drug Metab Toxicol.

[37] Schumacher-Schuh AF, Rieder CRM, Hutz MH (2014). Parkinson’s disease pharmacogenomics: new findings and perspectives. Pharmacogenomics.

[38] Fahn S, Oakes D, Shoulson I, Kieburtz K, Rudolph A, Lang A (2004). Levodopa and the progression of Parkinson’s disease. N Engl J Med.

[39] Kalinderi K, Fidani L, Katsarou Z, Bostantjopoulou S (2011). Pharmacological treatment and the prospect of pharmacogenetics in Parkinson’s disease. Int J Clin Pract.

[40] Ahlskog JE, Muenter MD (2001). Frequency of levodopa-related dyskinesias and motor fluctuations as estimated from the cumulative literature. Mov Disord.

[41] Nonnekes J, Timmer MHM, de Vries NM, Rascol O, Helmich RC, Bloem BR (2016). Unmasking levodopa resistance in Parkinson’s disease. Mov Disord.

[42] Pirtošek Z, Bajenaru O, Kovács N, Milanov I, Relja M, Skorvanek M (2020). Update on the management of Parkinson’s disease for general neurologists. Parkinsons Dis.

[43] Pistacchi M, Gioulis M, Sanson F, Marsala S (2017). Wearing off: A complex phenomenon often poorly recognized in Parkinson’s disease. A study with the WOQ-19 questionnaire. Neurol India.

[44] Olanow CW, Stern MB, Sethi K (2009). The scientific and clinical basis for the treatment of Parkinson disease. Neurology.

[45] Antonini A, Chaudhuri KR, Boroojerdi B, Asgharnejad M, Bauer L, Grieger F (2016). Impulse control disorder related behaviours during long-term rotigotine treatment: a post hoc analysis. Eur J Neurol.

[46] Gatto EM, Aldinio V (2019). Impulse control disorders in Parkinson’s Disease. A brief and comprehensive review. Front Neurol..

[47] Casu MA, Mocci I, Isola R, Pisanu A, Boi L, Mulas G (2020). Neuroprotection by the immunomodulatory drug pomalidomide in the Drosophila LRRK2WD40 genetic model of Parkinson’s disease. Front Aging Neurosci.

[48] Parsons CG (2019). CNS repurposing - potential new uses for old drugs: examples of screens for Alzheimer’s disease, Parkinson’s disease and spasticity. Neuropharmacology.

[49] Athauda D, Foltynie T (2018). Drug repurposing in Parkinson’s disease. CNS Drugs.

[50] Von Eichborn J, Murgueitio MS, Dunkel M, Koerner S, Bourne PE, Preissner R (2011). PROMISCUOUS: a database for network-based drug-repositioning. Nucleic Acids Res.

[51] Naylor S, Schonfeld JM (2014). Therapeutic drug repurposing, repositioning and rescue part i: overview. Drug Discovery World (DDW)..

[52] Talevi A, Bellera CL (2020). Challenges and opportunities with drug repurposing: finding strategies to find alternative uses of therapeutics. Expert Opin Drug Discov.

[53] Witkowski TX (2011). Intellectual property and other legal aspects of drug repurposing. Drug Discov Today Ther Strateg.

[54] Hernandez JJ, Pryszlak M, Smith L, Yanchus C, Kurji N, Shahani VM (2017). Giving drugs a second chance: overcoming regulatory and financial hurdles in repurposing approved drugs as cancer therapeutics. Front Oncol.

[55] Dudley JT, Deshpande T, Butte AJ (2011). Exploiting drug-disease relationships for computational drug repositioning. Brief Bioinform.

[56] Uenaka T, Satake W, Cha PC, Hayakawa H, Baba K, Jiang S (2018). In silico drug screening by using genome-wide association study data repurposed dabrafenib, an anti-melanoma drug, for Parkinson’s disease. Hum Mol Genet.

[57] Styczyńska-Soczka K, Zechini L, Zografos L (2017). Validating the predicted effect of astemizole and ketoconazole using a Drosophila model of Parkinson’s disease. Assay Drug Dev Technol.

[58] Siddiqi FH, Menzies FM, Lopez A, Stamatakou E, Karabiyik C, Ureshino R (2019). Felodipine induces autophagy in mouse brains with pharmacokinetics amenable to repurposing. Nat Commun.

[59] Poirier AA, Côté M, Bourque M, Morissette M, Di Paolo T, Soulet D (2016). Neuroprotective and immunomodulatory effects of raloxifene in the myenteric plexus of a mouse model of Parkinson’s disease. Neurobiol Aging.

[60] Ayoub BM, Mowaka S, Safar MM, Ashoush N, Arafa MG, Michel HE (2018). Repositioning of omarigliptin as a once-weekly intranasal anti-parkinsonian agent. Sci Rep.

[61] Fletcher EJR, Jamieson AD, Williams G, Doherty P, Duty S (2019). Targeted repositioning identifies drugs that increase fibroblast growth factor 20 production and protect against 6-hydroxydopamine-induced nigral cell loss in rats. Sci Rep.

[62] Rodriguez-Perez AI, Sucunza D, Pedrosa MA, Garrido-Gil P, Kulisevsky J, Lanciego JL (2018). Angiotensin type 1 receptor antagonists protect against alpha-synuclein-induced neuroinflammation and dopaminergic neuron death. Neurotherapeutics.

[63] Amireddy N, Puttapaka SN, Vinnakota RL, Ravuri HG, Thonda S, Kalivendi SV (2017). The unintended mitochondrial uncoupling effects of the FDA-approved anti-helminth drug nitazoxanide mitigates experimental parkinsonism in mice. J Biol Chem.

[64] Katila N, Bhurtel S, Shadfar S, Srivastav S, Neupane S, Ojha U (2017). Metformin lowers α-synuclein phosphorylation and upregulates neurotrophic factor in the MPTP mouse model of Parkinson’s disease. Neuropharmacology.

[65] Ozbey G, Nemutlu-Samur D, Parlak H, Yildirim S, Aslan M, Tanriover G (2020). Metformin protects rotenone-induced dopaminergic neurodegeneration by reducing lipid peroxidation. Pharmacol Rep.

[66] Simuni T, Fiske B, Merchant K, Coffey CS, Klingner E, Caspell-Garcia C (2021). Efficacy of nilotinib in patients with moderately advanced parkinson disease: a randomized clinical trial. JAMA Neurol.

[67] Son HJ, Han SH, Lee JA, Shin EJ, Hwang O (2017). Potential repositioning of exemestane as a neuroprotective agent for Parkinson’s disease. Free Radic Res.

[68] Mittal S, Bjørnevik K, Im DS, Flierl A, Dong X, Locascio JJ (2017). β2-Adrenoreceptor is a regulator of the α-synuclein gene driving risk of Parkinson’s disease. Science (80).

[69] Rinaldi F, Seguella L, Gigli S, Hanieh PN, Del Favero E, Cantù L (2019). inPentasomes: an innovative nose-to-brain pentamidine delivery blunts MPTP parkinsonism in mice. J Control Release.

[70] Bariotto dos Santos K, Padovan Neto FE, Bortolanza M, dos Santos Pereria M, Raisman-Vozari R, Tumas V (2019). Repurposing an established drug: an emerging role for methylene blue in L-DOPA-induced dyskinesia. Eur J Neurosci..

[71] Potts LF, Park ES, Woo JM, Dyavar Shetty BL, Singh A, Braithwaite SP (2015). Dual κ-agonist/μ-antagonist opioid receptor modulation reduces levodopa-induced dyskinesia and corrects dysregulated striatal changes in the nonhuman primate model of Parkinson disease. Ann Neurol.

[72] Bartlett MJ, Flores AJ, Ye T, Smidt SI, Dollish HK, Stancati JA (2020). Preclinical evidence in support of repurposing sub-anesthetic ketamine as a treatment for L-DOPA-induced dyskinesia. Exp Neurol.

[73] Lastres-Becker I, García-Yagüe AJ, Scannevin RH, Casarejos MJ, Kügler S, Rábano A (2016). Repurposing the NRF2 activator dimethyl fumarate as therapy against synucleinopathy in Parkinson’s disease. Antioxidants Redox Signal.

[74] Mahapatra A, Sarkar S, Biswas SC, Chattopadhyay K (2019). An aminoglycoside antibiotic inhibits both lipid-induced and solution-phase fibrillation of α-synuclein: In vitro. Chem Commun.

[75] González Lizárraga F, Ploper D, Ávila CL, Socías SB, dos Santos-Pereria M, Machín B (2020). CMT-3 targets different α-synuclein aggregates mitigating their toxic and inflammogenic effects. Sci Rep..

[76] González-Lizárraga F, Socías SB, Ávila CL, Torres-Bugeau CM, Barbosa LRS, Binolfi A (2017). Repurposing doxycycline for synucleinopathies: remodelling of α-synuclein oligomers towards non-toxic parallel beta-sheet structured species. Sci Rep.

[77] Marques CSF, Machado Júnior JB, de Andrade M, Andrade LN, Dos Santos ALS, Cruz E (2019). Use of pharmaceutical nanotechnology for the treatment of leishmaniasis. J Braz Soc Trop Med..

[78] Urrejola MC, Soto LV, Zumarán CC, Peñaloza JP, Álvarez B, Fuentevilla I (2018). Polymeric nanoparticle systems: structure, elaboration methods, characteristics, properties, biofunctionalization and self-assembly layer by layer technologies. Int J Morphol.

[79] Sharma G, Sharma AR, Lee SS, Bhattacharya M, Nam JS, Chakraborty C (2019). Advances in nanocarriers enabled brain targeted drug delivery across blood brain barrier. Int J Pharm.

[80] Gupta M, Lee HJ, Barden CJ, Weaver DF (2019). The blood-brain barrier (BBB) score. J Med Chem.

[81] Gallardo-Toledo E, Tapia-Arellano A, Celis F, Sinai T, Campos M, Kogan MJ (2020). Intranasal administration of gold nanoparticles designed to target the central nervous system: fabrication and comparison between nanospheres and nanoprisms. Int J Pharm.

[82] Ulbrich K, Knobloch T, Kreuter J (2011). Targeting the insulin receptor: nanoparticles for drug delivery across the blood–brain barrier (BBB). J Drug Target.

[83] Saraiva C, Praça C, Ferreira R, Santos T, Ferreira L, Bernardino L (2016). Nanoparticle-mediated brain drug delivery: overcoming blood–brain barrier to treat neurodegenerative diseases. J Control Release.

[84] Alavian F, Shams N (2020). Oral and intra-nasal administration of nanoparticles in the cerebral ischemia treatment in animal experiments: considering its advantages and disadvantages. Curr Clin Pharmacol.

[85] Chenthamara D, Subramaniam S, Ramakrishnan SG, Krishnaswamy S, Essa MM, Lin F-H (2019). Therapeutic efficacy of nanoparticles and routes of administration. Biomater Res.

[86] Pires A, Fortuna A, Alves G, Falcão A (2009). Intranasal drug delivery: how, why and what for?. J Pharm Pharm Sci.

[87] Reinholz J, Landfester K, Mailänder V (2018). The challenges of oral drug delivery via nanocarriers. Drug Deliv.

[88] Kaiser M, Pereira S, Pohl L, Ketelhut S, Kemper B, Gorzelanny C (2015). Chitosan encapsulation modulates the effect of capsaicin on the tight junctions of MDCK cells. Sci Rep.

[89] Lien CF, Molnár É, Toman P, Tsibouklis J, Pilkington GJ, Górecki DC (2012). In vitro assessment of alkylglyceryl-functionalized chitosan nanoparticles as permeating vectors for the Blood-Brain Barrier. Biomacromolecules.

[90] Bareford LM, Swaan PW (2007). Endocytic mechanisms for targeted drug delivery. Adv Drug Deliv Rev.

[91] Joanna R, Volker O, Inge SZ, Dick H (2004). Size-dependent internalization of particles via the pathways of clathrin- and caveolae-mediated endocytosis. Biochem J.

[92] Mendoza-Muñoz N, Urbán-Morlán Z, Leyva-Gómez G, De La Luz Z-Z, Piñón-Segundo E, Quintanar-Guerrero D (2021). Solid lipid nanoparticles: an approach to improve oral drug delivery. J Pharm Pharm Sci.

[93] Yuan H, Huang LF, Du YZ, Ying XY, You J, Hu FQ (2008). Solid lipid nanoparticles prepared by solvent diffusion method in a nanoreactor system. Colloids Surfaces B Biointerfaces.

[94] Desai MP, Labhasetwar V, Amidon GL, Levy RJ (1996). Gastrointestinal uptake of biodegradable microparticles: effect of particle size. Pharm Res.

[95] Yu M, Yang Y, Zhu C, Guo S, Gan Y (2016). Advances in the transepithelial transport of nanoparticles. Drug Discov Today.

[96] Bannunah AM, Vllasaliu D, Lord J, Stolnik S (2014). Mechanisms of nanoparticle internalization and transport across an intestinal epithelial cell model: effect of size and surface charge. Mol Pharm.

[97] Voigt N, Henrich-Noack P, Kockentiedt S, Hintz W, Tomas J, Sabel BA (2014). Surfactants, not size or zeta-potential influence blood-brain barrier passage of polymeric nanoparticles. Eur J Pharm Biopharm.

[98] Lombardo SM, Schneider M, Türeli AE, Türeli NG (2020). Key for crossing the BBB with nanoparticles: the rational design. Beilstein J Nanotechnol.

[99] Gao K, Jiang X (2006). Influence of particle size on transport of methotrexate across blood brain barrier by polysorbate 80-coated polybutylcyanoacrylate nanoparticles. Int J Pharm.

[100] Hughes JM, Budd PM, Tiede K, Lewis J (2015). Polymerized high internal phase emulsion monoliths for the chromatographic separation of engineered nanoparticles. J Appl Polym Sci.

[101] Del Prado-Audelo ML, Magaña JJ, Mejía-Contreras BA, Borbolla-Jiménez FV, Giraldo-Gomez DM, Piña-Barba MC (2019). In vitro cell uptake evaluation of curcumin-loaded PCL/F68 nanoparticles for potential application in neuronal diseases. J Drug Deliv Sci Technol.

[102] Shen S, Wu Y, Liu Y, Wu D (2017). High drug-loading nanomedicines: progress, current status, and prospects. Int J Nanomedicine.

[103] Shen Y, Jin E, Zhang B, Murphy CJ, Sui M, Zhao J (2010). Prodrugs forming high drug loading multifunctional nanocapsules for intracellular cancer drug delivery. J Am Chem Soc.

[104] Nigam K, Kaur A, Tyagi A, Nematullah M, Khan F, Gabrani R (2019). Nose-to-brain delivery of lamotrigine-loaded PLGA nanoparticles. Drug Deliv Transl Res.

[105] Deepika MS, Thangam R, Sheena TS, Vimala RTV, Sivasubramanian S, Jeganathan K (2019). Dual drug loaded PLGA nanospheres for synergistic efficacy in breast cancer therapy. Mater Sci Eng C.

[106] Bhakay A, Rahman M, Dave RN, Bilgili E (2018). Bioavailability enhancement of poorly water-soluble drugs via nanocomposites: formulation-processing aspects and challenges. Pharmaceutics.

[107] Kocbek P, Baumgartner S, Kristl J (2006). Preparation and evaluation of nanosuspensions for enhancing the dissolution of poorly soluble drugs. Int J Pharm.

[108] Aggarwal P, Hall JB, McLeland CB, Dobrovolskaia MA, McNeil SE (2009). Nanoparticle interaction with plasma proteins as it relates to particle biodistribution, biocompatibility and therapeutic efficacy. Adv Drug Deliv Rev.

[109] Roach P, Farrar D, Perry CC (2005). Interpretation of protein adsorption: surface-induced conformational changes. J Am Chem Soc.

[110] Owens DE, Peppas NA (2006). Opsonization, biodistribution, and pharmacokinetics of polymeric nanoparticles. Int J Pharm.

[111] Gessner A, Lieske A, Paulke BR, Müller RH (2002). Influence of surface charge density on protein adsorption on polymeric nanoparticles: analysis by two-dimensional electrophoresis. Eur J Pharm Biopharm.

[112] Cedervall T, Lynch I, Lindman S, Berggård T, Thulin E, Nilsson H (2007). Understanding the nanoparticle-protein corona using methods to quntify exchange rates and affinities of proteins for nanoparticles. Proc Natl Acad Sci U S A.

[113] Pinelli F, Perale G, Rossi F (2020). Coating and functionalization strategies for nanogels and nanoparticles for selective drug delivery. Gels.

[114] Du W, Fan Y, Zheng N, He B, Yuan L, Zhang H (2013). Transferrin receptor specific nanocarriers conjugated with functional 7peptide for oral drug delivery. Biomaterials.

[115] Tang S, Wang A, Yan X, Chu L, Yang X, Song Y (2019). Brain-targeted intranasal delivery of dopamine with borneol and lactoferrin co-modified nanoparticles for treating Parkinson’s disease. Drug Deliv.

[116] Leveugle B, Faucheux BA, Bouras C, Nillesse N, Spik G, Hirsch EC (1996). Cellular distribution of the iron-binding protein lactotransferrin in the mesencephalon of Parkinson’s disease cases. Acta Neuropathol.

[117] Lopalco A, Cutrignelli A, Denora N, Lopedota A, Franco M, Laquintana V (2018). Transferrin functionalized liposomes loading dopamine HCl: development and permeability studies across an in vitro model of human blood-brain barrier. Nanomaterials.

[118] Kang YS, Jung HJ, Oh JS, Song DY (2016). Use of PEGylated immunoliposomes to deliver dopamine across the blood-brain barrier in a rat model of Parkinson’s disease. CNS Neurosci Ther.

[119] Sridhar V, Gaud R, Bajaj A, Wairkar S (2018). Pharmacokinetics and pharmacodynamics of intranasally administered selegiline nanoparticles with improved brain delivery in Parkinson’s disease. Nanomed Nanotechnol Biol Med.

[120] Hu K, Chen X, Chen W, Zhang L, Li J, Ye J (2018). Neuroprotective effect of gold nanoparticles composites in Parkinson’s disease model. Nanomed Nanotechnol Biol Med.

[121] Huang R, Ma H, Guo Y, Liu S, Kuang Y, Shao K (2013). Angiopep-conjugated nanoparticles for targeted long-term gene therapy of parkinson’s disease. Pharm Res.

[122] Ray S, Sinha P, Laha B, Maiti S, Bhattacharyya UK, Nayak AK (2018). Polysorbate 80 coated crosslinked chitosan nanoparticles of ropinirole hydrochloride for brain targeting. J Drug Deliv Sci Technol.

[123] Zhao Y, Xiong S, Liu P, Liu W, Wang Q, Liu Y (2020). Polymeric nanoparticles-based brain delivery with improved therapeutic efficacy of ginkgolide B in Parkinson’s disease. Int J Nanomedicine.

[124] Shadab MD, Khan RA, Mustafa G, Chuttani K, Baboota S, Sahni JK (2013). Bromocriptine loaded chitosan nanoparticles intended for direct nose to brain delivery: pharmacodynamic, pharmacokinetic and scintigraphy study in mice model. Eur J Pharm Sci.

[125] Gambaryan PY, Kondrasheva IG, Severin ES, Guseva AA, Kamensky AA (2014). Increasing the effciency of parkinson’s disease treatment using a poly(lactic-co-glycolic acid) (PLGA) based L-DOPA delivery system. Exp Neurobiol.

[126] Fernandes C, Martins C, Fonseca A, Nunes R, Matos MJ, Silva R (2018). PEGylated PLGA nanoparticles as a smart carrier to increase the cellular uptake of a coumarin-based monoamine oxidase B inhibitor. ACS Appl Mater Interfaces.

[127] Huang R, Han L, Li J, Ren F, Ke W, Jiang C (2009). Neuroprotection in a 6-hydroxydopamine-lesioned Parkinson model using lactoferrin-modified nanoparticles. J Gene Med.

[128] Song Y, Shi Y, Zhang L, Hu H, Zhang C, Yin M (2019). Synthesis of CSK-DEX-PLGA nanoparticles for the oral delivery of exenatide to improve its mucus penetration and intestinal absorption. Mol Pharm.

[129] Kandilli B, Ugur Kaplan AB, Cetin M, Taspinar N, Ertugrul MS, Aydin IC (2020). Carbamazepine and levetiracetam-loaded PLGA nanoparticles prepared by nanoprecipitation method: in vitro and in vivo studies. Drug Dev Ind Pharm.

[130] CN104055735A. Semaglutide liposome and preparation method thereof. 2013. p. 1–21. https://patents.google.com/patent/CN104055735A/en. Accessed 28 Feb 2021.

[131] Liu G, Yang J, Wang Y, Liu X, Guan LL, Chen L (2019). Protein-lipid composite nanoparticles for the oral delivery of vitamin B 12: impact of protein succinylation on nanoparticle physicochemical and biological properties. Food Hydrocoll.

[132] Sadozai SK, Khan SA, Karim N, Becker D, Steinbrück N, Gier S (2020). Ketoconazole-loaded PLGA nanoparticles and their synergism against *Candida*
*albicans* when combined with silver nanoparticles. J Drug Deliv Sci Technol.

[133] Jana U, Mohanty AK, Pal SL, Manna PK, Mohanta GP (2014). Felodipine loaded PLGA nanoparticles: preparation, physicochemical characterization and in vivo toxicity study. Nano Converg.

[134] He Y, Zhan C, Pi C, Zuo Y, Yang S, Hu M (2020). Enhanced oral bioavailability of felodipine from solid lipid nanoparticles prepared through effervescent dispersion technique. AAPS PharmSciTech.

[135] Saini D, Fazil M, Ali MM, Baboota S, Ali J (2015). Formulation, development and optimization of raloxifene-loaded chitosan nanoparticles for treatment of osteoporosis. Drug Deliv.

[136] Ravi PR, Aditya N, Kathuria H, Malekar S, Vats R (2014). Lipid nanoparticles for oral delivery of raloxifene: optimization, stability, in vivo evaluation and uptake mechanism. Eur J Pharm Biopharm.

[137] Dudhipala N, Veerabrahma K (2016). Candesartan cilexetil loaded solid lipid nanoparticles for oral delivery: characterization, pharmacokinetic and pharmacodynamic evaluation. Drug Deliv.

[138] ÖztÜrk N, Kara A, Vural İ (2020). Formulation and in vitro evaluation of telmisartan nanoparticles prepared by emulsion-solvent evaporation technique. Turkish J Pharm Sci.

[139] Abbasalipourkabir R, Fallah M, Sedighi F, Maghsood AH, Javid S (2016). Nanocapsulation of nitazoxanide in solid lipid nanoparticles as a new drug delivery system and in vitro release study. J Biol Sci.

[140] Kumar S, Bhanjana G, Verma RK, Dhingra D, Dilbaghi N, Kim K-H (2017). Metformin-loaded alginate nanoparticles as an effective antidiabetic agent for controlled drug release. J Pharm Pharmacol.

[141] Archibald M, Pritchard T, Nehoff H, Rosengren RJ, Greish K, Taurin S (2016). A combination of sorafenib and nilotinib reduces the growth of castrate-resistant prostate cancer. Int J Nanomedicine.

[142] Jayapal JJ, Dhanaraj S (2017). Exemestane loaded alginate nanoparticles for cancer treatment: formulation and in vitro evaluation. Int J Biol Macromol.

[143] Beck-Broichsitter M, Gauss J, Gessler T, Seeger W, Kissel T, Schmehl T (2010). Pulmonary targeting with biodegradable salbutamol-loaded nanoparticles. J Aerosol Med Pulm Drug Deliv.

[144] Valle IV, Machado ME, Araujo CDCB, Da Cunha-Junior EF, Da Silva PJ, Torres-Santos EC (2019). Oral pentamidine-loaded poly(d, l-lactic-co-glycolic) acid nanoparticles: an alternative approach for leishmaniasis treatment. Nanotechnology.

[145] Omarch G, Kippie Y, Mentor S, Ebrahim N, Fisher D, Murilla G (2019). Comparative in vitro transportation of pentamidine across the blood-brain barrier using polycaprolactone nanoparticles and phosphatidylcholine liposomes. Artif Cells Nanomed Biotechnol.

[146] Manimekalai P, Dhanalakshmi R, Manavalan R (2017). Preparation and characterization of ceftriaxone sodium encapsulated chitosan nanoparticles. Int J Appl Pharm.

[147] Gattani SG, Moon RS (2018). Formulation and evaluation of fast dissolving tablet containing vilazodone nanocrystals for solubility and dissolution enhancement using soluplus: in vitro-in vivo study. J Appl Pharm Sci.

[148] Jesus VPS, Raniero L, Lemes GM, Bhattacharjee TT, Caetano Júnior PC, Castilho ML (2018). Nanoparticles of methylene blue enhance photodynamic therapy. Photodiagnosis Photodyn Ther.

[149] Khanna K, Sharma N, Rawat S, Khan N, Karwasra R, Hasan N (2021). Intranasal solid lipid nanoparticles for management of pain: a full factorial design approach, characterization & gamma scintigraphy. Chem Phys Lipids.

[150] Han FY, Liu Y, Kumar V, Xu W, Yang G, Zhao CX (2020). Sustained-release ketamine-loaded nanoparticles fabricated by sequential nanoprecipitation. Int J Pharm.

[151] Kumar P, Sharma G, Kumar R, Malik R, Singh B, Katare OP (2017). Enhanced brain delivery of dimethyl fumarate employing tocopherol-acetate-based nanolipidic carriers: evidence from pharmacokinetic, biodistribution, and cellular uptake studies. ACS Chem Neurosci.

[152] Payne JN, Waghwani HK, Connor MG, Hamilton W, Tockstein S, Moolani H (2016). Novel synthesis of kanamycin conjugated gold nanoparticles with potent antibacterial activity. Front Microbiol.

[153] Misra R, Sahoo SK (2012). Antibacterial activity of doxycycline-loaded nanoparticles. Methods Enzymol.

